# Chromosome-scale reference genome of an ancient landrace: unveiling the genetic basis of seed weight in the food legume crop pigeonpea (*Cajanus cajan*)

**DOI:** 10.1093/hr/uhae201

**Published:** 2024-07-30

**Authors:** Chun Liu, Xipeng Ding, Yuanhang Wu, Jianyu Zhang, Rui Huang, Xinyong Li, Guodao Liu, Pandao Liu

**Affiliations:** Tropical Crops Genetic Resources Institute, National Key Laboratory for Tropical Crop Breeding, Chinese Academy of Tropical Agricultural Sciences, Haikou/Sanya 571101/572024, China; Key Laboratory of Crop Gene Resources and Germplasm Enhancement in Southern China, Ministry of Agriculture and Rural Affairs, Haikou 571101, China; Key Laboratory of Tropical Crops Germplasm Resources Genetic Improvement and Innovation of Hainan Province, Haikou 571101, China; School of Tropical Agriculture and Forestry, Sanya Institute Breeding and Multiplication, Hainan University, Haikou/Sanya 570228/572025, China; Tropical Crops Genetic Resources Institute, National Key Laboratory for Tropical Crop Breeding, Chinese Academy of Tropical Agricultural Sciences, Haikou/Sanya 571101/572024, China; School of Nuclear Technology and Chemistry & Biology, Hubei University of Science and Technology, Xianning 437100, China; School of Tropical Agriculture and Forestry, Sanya Institute Breeding and Multiplication, Hainan University, Haikou/Sanya 570228/572025, China; Tropical Crops Genetic Resources Institute, National Key Laboratory for Tropical Crop Breeding, Chinese Academy of Tropical Agricultural Sciences, Haikou/Sanya 571101/572024, China; Tropical Crops Genetic Resources Institute, National Key Laboratory for Tropical Crop Breeding, Chinese Academy of Tropical Agricultural Sciences, Haikou/Sanya 571101/572024, China; Tropical Crops Genetic Resources Institute, National Key Laboratory for Tropical Crop Breeding, Chinese Academy of Tropical Agricultural Sciences, Haikou/Sanya 571101/572024, China; Tropical Crops Genetic Resources Institute, National Key Laboratory for Tropical Crop Breeding, Chinese Academy of Tropical Agricultural Sciences, Haikou/Sanya 571101/572024, China; Key Laboratory of Crop Gene Resources and Germplasm Enhancement in Southern China, Ministry of Agriculture and Rural Affairs, Haikou 571101, China; Key Laboratory of Tropical Crops Germplasm Resources Genetic Improvement and Innovation of Hainan Province, Haikou 571101, China

## Abstract

Pigeonpea (*Cajanus cajan*) is a nutrient-rich and versatile food legume crop of tropical and subtropical regions. In this study, we describe the *de novo* assembly of a high-quality genome for the ancient pigeonpea landrace ‘D30’, achieved through a combination of Pacific Biosciences high-fidelity (PacBio HiFi) and high-throughput chromatin conformation capture (Hi-C) sequencing technologies. The assembled ‘D30’ genome has a size of 813.54 Mb, with a contig N50 of 10.74 Mb, a scaffold N50 of 73.07 Mb, and a GC content of 35.67%. Genomic evaluation revealed that the ‘D30’ genome contains 99.2% of Benchmarking Universal Single-Copy Orthologs (BUSCO) and achieves a 29.06 long terminal repeat (LTR) assembly index (LAI). Genome annotation indicated that ‘D30’ encompasses 431.37 Mb of repeat elements (53.02% of the genome) and 37 977 protein-coding genes. Identification of single-nucleotide polymorphisms (SNPs), insertions/deletions (indels), and structural variations between ‘D30’ and the published genome of pigeonpea cultivar ‘Asha’ suggests that genes affected by these variations may play important roles in biotic and abiotic stress responses. Further investigation of genomic regions under selection highlights genes enriched in starch and sucrose metabolism, with 42.11% of these genes highly expressed in seeds. Finally, we conducted genome-wide association studies (GWAS) to facilitate the identification of 28 marker–trait associations for six agronomic traits of pigeonpea. Notably, we discovered a calmodulin-like protein (*CcCML*) that harbors a dominant haplotype associated with the 100-seed weight of pigeonpea. Our study provides a foundational resource for developing genomics-assisted breeding programs in pigeonpea.

## Introduction

Pigeonpea (*Cajanus cajan*) is the sixth most important food legume crop, with cultivation spanning ~7 million hectares (ha) worldwide [1]. Compared with other legume crops, pigeonpea exhibits superior productivity in adverse environmental conditions, such as high temperatures, drought, aluminum toxicity, and nutrient-poor soils [2–4]. This makes it an ideal choice for cultivation by smallholder farmers in developing regions of Asia, Africa, and the tropical Americas, serving as a main source of protein and income for them [5]. Apart from its use as a food source, pigeonpea also serves various other purposes, including as livestock fodder, green manure, domestic firewood, and for medicinal applications [2, 6, 7]. Its multiple uses and low input requirements render it a sustainable crop in marginal environments, offering significant promise in addressing food security and nutritional needs in tropical and subtropical regions [8].

Belonging to the millettioid (tropical) clade within the tribe Phaseoleae, pigeonpea shares this botanical grouping with legume crop species like soybean (*Glycine max*) and common bean (*Phaseolus vulgaris*). The domestication of pigeonpea, which began around 3500 years ago in central India from its wild progenitor *Cajanus cajanifolius*, led to the development of diverse landraces within the region [8]. These landraces were later spread for cultivation in various geographical regions, with some being transported to over 100 countries by traders and migrant workers [9]. Despite the importance of pigeonpea, genomic research has been hampered by the limited availability of high-quality reference genomes. The published genome of the pigeonpea cultivar ‘Asha’, assembled using second-generation sequencing [10], falls short of providing the comprehensive genetic insights required for genomics-assisted breeding programs. Although there have been some genomic updates, such as genome-wide association studies (GWAS), pan-genome analysis, and superior haplotype analysis based on the ‘Asha’ genome [6, 8, 11, 12], a high-quality reference genome for pigeonpea is still lacking, hindering further advancements in genetic research.

**Figure 1:**
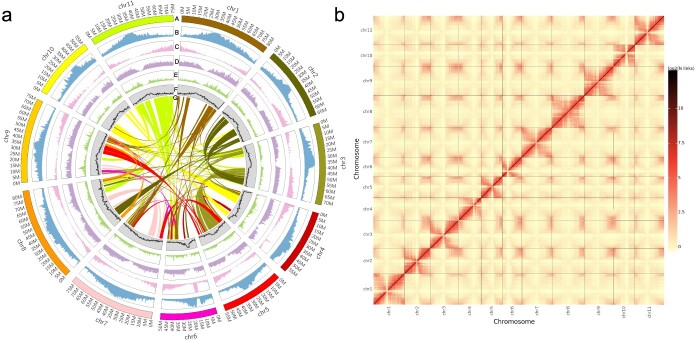
Genomic features of pigeonpea landrace ‘D30’ and heat map of Hi-C chromosomal interactions. **a** Features of the assembled ‘D30’ genome. (A) chromosomes of the ‘D30’ genome. (B) Repeat element density. (C) Gene density. (D) Variant density (including SNPs and indels) in ‘Asha’ compared with ‘D30’. (E) Variant density of 294 *Cajanus* accessions. (F) GC content. (G) Intraspecific collinearity between chromosomes. The contents of B–F were calculated using a non-overlapping window size of 500 kb. **b** Hi-C interactions among 11 chromosomes of the ‘D30’ genome. Dark red indicates strong interactions and yellow indicates weak interactions.

Wild relatives or ancient landraces are indispensable for genomic and genetic research in various species, offering a wealth of genetic diversity and traits that can be harnessed for the improvement of cultivated crops. It has been reported that wild variants can contribute to improved nitrogen-use efficiency and seed protein content in maize (*Zea mays*) [13]. The wild tea tree DASZ (*Camellia sinensis*) genome provides insights into the pedigree and selection history of cultivated tea varieties, highlighting how wild species inform breeding practices [14]. A novel salt tolerance gene was identified in wild soybean (*G. max*), highlighting the potential of wild species in contributing genes for stress tolerance [15]. At least six wild relatives of pigeonpea originated in China, including *Cajanus crassus*, *C. goensis*, *C. grandiflorus*, *C. mollis*, *C. niveus*, and *C. scarabaeoides* [16]. Furthermore, in southern China smallholder farmers have cultivated pigeonpea for over 1500 years, resulting in the emergence of a considerable number of landraces [17].

In this study, we introduce an ancient Chinese landrace of pigeonpea named ‘D30’ and perform a *de novo* chromosome-scale assembly of its genome. This was accomplished using the state-of-the-art Pacific Biosciences (PacBio) HiFi sequencing technique, high-throughput chromatin conformation capture (Hi-C) sequencing, and next-generation sequencing (NGS). Subsequently, we identified genetic variation loci underlying agronomic traits of pigeonpea through GWAS. Our study not only fills the gap in the lack of a high-quality reference genome for pigeonpea but also lays the foundation for the genetic improvement of existing pigeonpea cultivars.

## Results

### Genome sequencing and assembly of pigeonpea landrace ‘D30’

The genome of the Chinese landrace ‘D30’ of pigeonpea was sequenced and assembled by combining state-of-the-art technologies, including PacBio HiFi, Hi-C, and NGS. (Fig. 1a; Table 1). By employing *k*-mer analysis on the NGS reads (41.08 Gb clean data; Supplementary Data Table S1), the genome size of ‘D30’ was estimated to be 823.55 Mb (Supplementary Data Fig. S1). Additionally, 27.4 Gb (~33.35× coverage; Supplementary Data Table S2) of PacBio HiFi reads were generated and assembled into 1728 high-quality contigs using hifiasm. Subsequently, 180 contigs were organized into 11 chromosomes, utilizing 101.52 Gb (~123.35× coverage; Supplementary Data Table S3) of Hi-C reads (Fig. 1b). Ultimately, the assembled ‘D30’ genome size reached 813.54 Mb, with a contig N50 of 10.74 Mb and a scaffold N50 of 73.07 Mb. Approximately 92.82% of the genome sequences were anchored to 11 chromosomes (Table 1; Fig. 1). The assembled genome constituted 98.78% of the estimated genome size. The quality of the genome assembly was evaluated using Benchmarking Universal Single-Copy Orthologs (BUSCO) and the long terminal repeat (LTR) assembly index (LAI), yielding a BUSCO completeness of 99.2% (95.7% single-copy and 3.5% duplicated orthologs) and an LAI of 29.06 (Table 1; Supplementary Data Table S4). The completeness of the assembled genome was further verified by remapping the PacBio HiFi and NGS reads, revealing that >98.26% of HiFi reads and 97.39% of NGS reads aligned accurately. This indicates a high degree of completeness for the assembled genome. In terms of genomic integrity and continuity, the assembled ‘D30’ genome exhibited significant superiority over previously assembled pigeonpea genomes (Table 1).

**Table 1:** Statistical and comparative analysis of genomic information of pigeonpea.

	**‘D30’ (this study)**	**‘Asha’ [10]**	**‘Asha’ [23]**
Estimated genome size (Mb)	823.55	833	833
Assembly genome size (Mb)	813.54	605.78	594.8
Number of contigs	1 728	173 959	76 499
Contig N50 (Mb)	10.74	0.008	0.021
Scaffold N50 (Mb)	73.07	0.52	53.9
Longest contig (Mp)	42.48	0.19	0.19
GC content (%)	35.67	32.82	32.79
BUSCO (%)	99.2	97.9	97.8
LAI	29.06	4.29	5.27
Number of chromosomes	11	11	11
Anchored chromosome length (Mb)	755.14	247.49	543.34
Chromosome anchored ratio (%)	92.82	40.86	91.35
Repeat content (Mb)	431.37	302	293.11
Repeat ratio (%)	53.02	49.95	49.28
Number of protein-coding genes	37 977	48 680	29 482
Mean exon length (bp)	226.14	267.39	223.79
Mean intron length (bp)	606.24	536.89	638.6

### Annotation of the assembled genome of pigeonpea landrace ‘D30’

The ‘D30’ genome contained 431.37 Mb of repeat elements (53.02% of the genome), a proportion comparable to those observed in soybean and common bean (Fig. 1a; Table 1; Supplementary Data Table S5). The majority of repeat sequences in pigeonpea were composed of LTRs (372.76 Mb), representing 45.82% of the assembled genome, a proportion that was higher than those in soybean (39.71%), common bean (40.60%), and medicago (*Medicago truncatula*) (18.19%) (Supplementary Data Table S5). Gypsy and Copia elements, types of LTR retrotransposons, comprised 36.82 and 8.44% of the pigeonpea genome, respectively. Furthermore, the proportion of Gypsy was found to be greater in pigeonpea than in soybean (28.22%), common bean (27.16%), and medicago (9.39%) (Supplementary Data Table S5). Significantly, we identified more than twice as many intact LTRs in pigeonpea (12927) compared with soybeans (4 522) and common beans (5 074), with 79.41% of these intact LTRs occurring recently in the pigeonpea genome, evidenced by insertion times that were less than 1 million years ago (MYA) (Supplementary Data Fig. S2). Additionally, we found that 4.80% of the intact LTRs resided within gene bodies, extending the average intron and gene lengths of these 586 genes (Supplementary Data Fig. S3).

To predict protein-coding genes, transcriptomes from roots, stems, buds, leaves, pods, and seeds of ‘D30’ were sequenced using RNA-seq, yielding a total of 42.23 Gb (~7.04 Gb per sample) of clean data (Supplementary Data Table S6). Subsequently, the clean data were mapped onto the ‘D30’ genome for transcript construction. We identified 37 977 protein-coding genes through a combination of *ab initio* prediction, homology-based prediction, and transcript evidence (Fig. 1a; Table 1). The average lengths of exons and introns in the predicted genes were 226.14 and 606.24 bp, respectively (Table 1; Supplementary Data Table S7). The number of predicted genes for ‘D30’ was similar to that of *Medicago polymorpha* (36 087 predicted genes) [18] and *M. truncatula* (44 623 predicted genes) [19], but fewer than that of *G. max* (55 498 predicted genes) [20]. The BUSCO completeness of the predicted genes was 97.6%, with only 1% of Embryophyta orthologs either unassembled or unannotated in the assembled genome, indicating a high level of completeness in the annotated gene set (Supplementary Data Table S8). Functional annotation revealed that 93.39% of the predicted genes received annotations, with 24 897 (65.56%) and 24 890 (65.54%) genes assigned to the KEGG and GO databases, respectively (Supplementary Data Table S9). Additionally, read counts and transcripts per million (TPM) calculations revealed that a total of 33 095 genes were expressed across various tissues. Moreover, a total of 174 microRNAs (miRNAs), 3 035 transfer RNAs (tRNAs), 5 645 ribosomal RNAs (rRNAs), and 931 small nuclear RNAs (snRNAs) were predicted in the pigeonpea genome (Supplementary Data Table S10).

### Variations between pigeonpea landrace ‘D30’ and cultivar ‘Asha’

**Figure 2:**
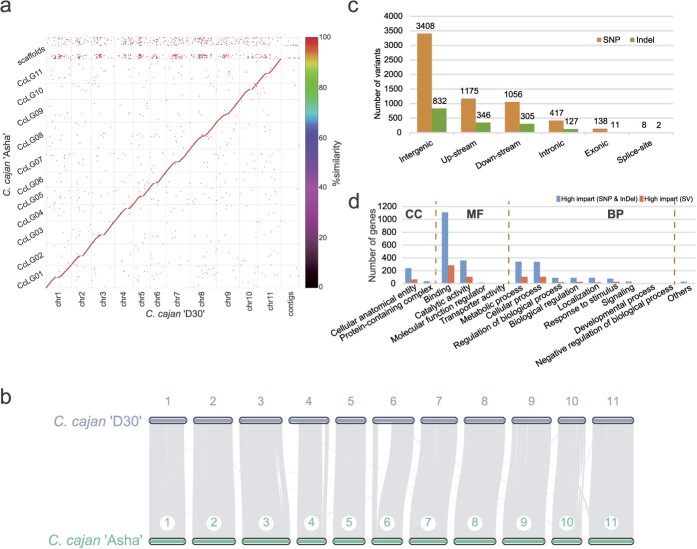
Whole-genome comparison between pigeonpea cultivar ‘Asha’ and landrace ‘D30’. **a** Genomic synteny comparisons. Colors represent the identity of alignment. **b** Gene synteny comparisons. **c** Genomic location of SNPs and indels identified in ‘Asha’ compared with ‘D30’. **d** GO terms of high-impact genes affected by SNPs, indels, and SVs.

Whole-genome alignment and gene collinearity analysis results showed substantial collinearity between the ‘D30’ and ‘Asha’ genomes, although there were some structural variations (SVs), including an inversion on chromosome 10 (Fig. 2a and b). Given that the ‘Asha’ genome assembly was derived from NGS reads, resulting in a smaller genome size than estimated, a read-based mapping approach was employed to identify single-nucleotide polymorphisms (SNPs), insertions/deletions (indels), and SVs between the ‘Asha’ and ‘D30’ genomes. A total of 3.97 million SNPs and 0.97 million indels were identified in the ‘Asha’ genome. The majority of these variations were found in intergenic regions (54.95% for SNPs and 51.24% for indels), followed by regions upstream and downstream of genes (Fig. 2c). Approximately 2.22% of SNPs and 0.70% of indels were located in exon regions of genes, categorizing these variants as high-impact variants (Fig. 2c). More importantly, 4 010 high-impact SNPs and 7 470 high-impact indels were identified, affecting 5 725 genes. Most of these high-impact genes were associated with the GO terms ‘binding’ and ‘catalytic activity’ for molecular function, as well as ‘metabolic process’ and ‘cellular process’ for biological process (Fig. 2d). Detailed functional annotation indicated that these high-impact genes included 386 R genes (Supplementary Data Table S11), which were potentially involved in the response to abiotic stress.

Additionally, PacBio sequencing reads of ‘Asha’ were mapped onto the ‘D30’ genome to identify SVs. In comparison with the ‘D30’ genome, 63 473 homozygous and 17 575 heterozygous SVs were identified in the ‘Asha’ genome, corroborated by at least two detection methods (Supplementary Data Table S12). Deletions and insertions, constituting 50.30 and 49.53%, respectively, emerged as the dominant SVs among the homozygous variants (Supplementary Data Table S12). Among these deletions, 1 575 overlapped with the coding region (high-impact SVs), affecting 1885 genes. The majority of these genes were associated with the GO terms ‘binding’ and ‘catalytic activity’ for molecular function, as well as ‘metabolic process’ and ‘cellular process’ for biological process, similar to the genes affected by SNPs and indels (Fig. 2d). Further GO enrichment analysis showed significant enrichment in these genes for the GO term ‘response to high light intensity’ (FDR < 0.05; Supplementary Data Table S13). Detailed functional annotation indicated that these high-impact genes contained 74 R genes (Supplementary Data Table S14). Genes affected by these high-impact SVs may play important roles in biotic and abiotic stress response.

### Comparative genomic analyses of pigeonpea and other plant species

**Figure 3:**
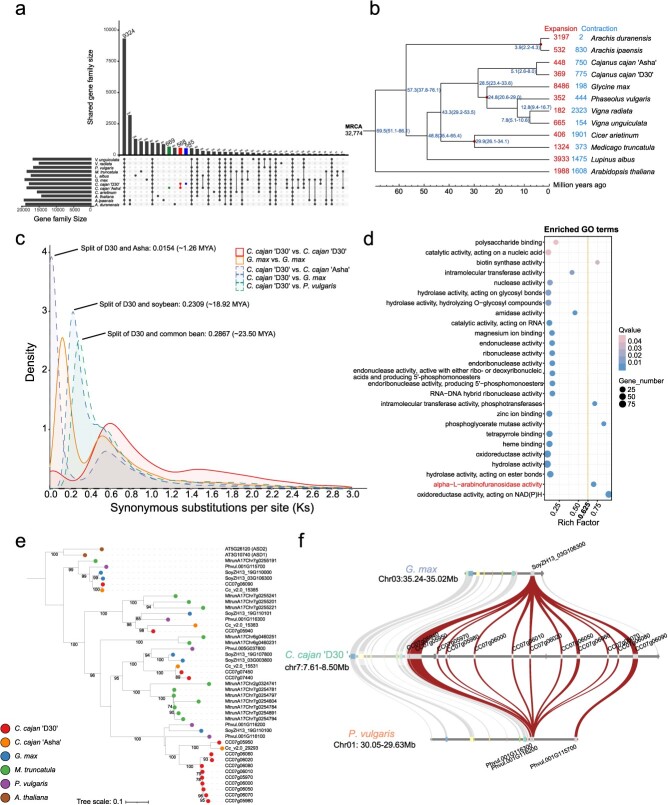
Comparative genomics of pigeonpea and other plant species. **a** OGs and shared OGs of studied Fabaceae species and arabidopsis. **b** Phylogenetic trees, divergence time, and expansion–contraction analysis of studied species based on single-copy orthologous groups. **c**  *K*_s_ distribution of collinear gene pairs within and between pigeonpea, soybean, and common bean. **d** GO enrichment analysis of genes from ‘D30’-specific and ‘D30’-expanded OGs. **e** Phylogenetic trees of alpha-l-arabinofuranosidase 1 (*ASD1*) in pigeonpea, soybean, common bean, and arabidopsis. **f** Microcollinearity of *ASD1* genes in ‘D30’ compared with soybean and common bean. Red curve represents *ASD1* genes.

To explore the evolution of pigeonpea, its genome was compared with those of 10 other Fabaceae species and arabidopsis (*Arabidopsis thaliana*). Protein sequences from these genomes were clustered into 32 774 orthologous groups (OGs), with 9 324 OGs shared across all studied species and 568 OGs shared only by ‘D30’ and ‘Asha’ (Fig. 3a). Phylogenetic trees were constructed and divergence time was estimated based on 561 single-copy OGs. The constructed phylogenetic tree confirmed that pigeonpea was located in the millettioid clade within the subfamily Papilionoideae, which includes soybean and common bean, the important legume crop species (Fig. 3b). Furthermore, contraction and expansion of the OGs were performed, and identified 545 ‘D30’-specific OGs and 369 ‘D30’-expanded OGs (Fig. 3b). GO enrichment analysis showed that these genes were significantly enriched in terms of ‘oxidoreductase activity, acting on NAD(P)H’ (GO:0016651), ‘alpha-l-arabinofuranosidase activity’ (GO:0046556), ‘phosphoglycerate mutase activity’ (GO:0004619), ‘biotin synthase activity’ (GO:0004076), etc. (Fig. 3d). Gene identification and comparison highlighted that there were more copy number of alpha-l-arabinofuranosidase 1 (*ASD1*) in ‘D30’ compared with soybean and common bean, which were expanded mainly through tandem duplication (Fig. 3e and f; Supplementary Data Table S15). Notably, the *ASD1* genes in ‘D30’ exhibited significant tissue-specific expression (Supplementary Data Fig. S4), indicating potential functional differentiation.

**Figure 4:**
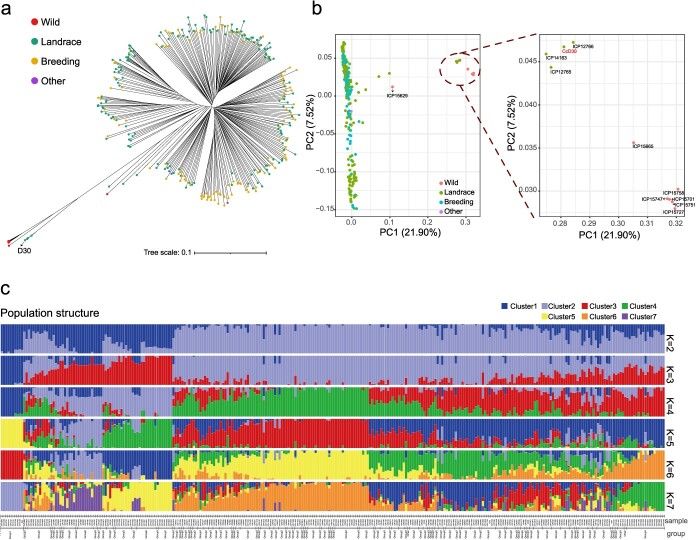
Population analysis of 294 *Cajanus* accessions: including ‘D30’ from this study, ‘Asha’ [10], and 292 other *Cajanus* accessions [8] from published research. **a** Phylogenetic tree of the *Cajanus* accessions. **b** PCA of the *Cajanus* accessions. **c** Population structure analysis of the *Cajanus* accessions. In the grouping information at the bottom, Group 1 was categorized based on the population phylogenetic tree, PCA, and population structure, while landraces not included in Group 1 were designated as Group 2, and breeding lines were designated as Group 3.

Moreover, we identified collinear gene blocks within and between pigeonpea, soybean, and common bean, and calculated synonymous substitution rates per site (*K*_s_) for each collinear gene pair. The results confirmed the absence of a recent whole-genome duplication event in pigeonpea, which was consistent with previously reported findings [10]. Furthermore, our analysis determined that the split of ‘D30’ and ‘Asha’ occurred at ~1.26 MYA, while the split of pigeonpea, soybean, and common bean occurred at ~18.92 and 23.50 MYA, respectively (Fig. 3c).

### Population structure and selection signals in the improvement of pigeonpea

Whole-genome sequencing (WGS) data for 292 *Cajanus* accessions were retrieved from the NCBI Sequence Read Archive (SRA) under BioProject accession number PRJNA383013, as previously reported [8]. Resequencing data of 292 *Cajanus* accessions, along with ‘Asha’ and ‘D30’, were mapped onto the ‘D30’ genome for the analysis of population variations. A total of 2.1 million high-quality variants were identified across the 294 accessions, including 1.71 million SNPs and 0.39 million indels. More than half of the variations resided in the intergenic region (56.61% for SNPs and 54.18% for indels), while variations within the exon region comprised 2.98% of SNPs and 1.16% of indels, respectively (Supplementary Data Table S16). The construction of a phylogenetic tree for the *Cajanus* accessions revealed that ‘D30’ was closely related to three landraces and six wild species (termed Group 1) (Fig. 4a and c). Subsequent principal component analysis (PCA) also indicated that PC1 (which explained 21.90% of the variance) and PC2 (which explained 7.52% of the variance) could clearly distinguish between Group 1 and other accessions (Fig. 4b). ‘D30’ fell within the range of Group 1 and was closely related to three landraces: ICP12766, ICP14163, and ICP12765 (Fig. 4b). We varied the number of presumed ancestral populations (*K*, from 2 to 10) to identify genetically distinct clusters. We observed that none of the *K* values ranging from 2 to 10 showed minimal cross-validation (CV) error (Supplementary Data Fig. S5). Given the clear clustering of the population phylogenetic tree into seven groups, we proceeded with the results obtained from *K* values of 2 to 7 for subsequent analyses. When *K* > 5, Group 1 formed a unique cluster, displaying a distinct population structure compared with other accessions, suggesting that ‘D30’ and these three landraces might be ancient pigeonpea accessions (Fig. 4c). Consequently, the landraces not included in Group 1 were designated as Group 2, and breeding lines were designated as Group 3, for further linkage disequilibrium (LD) and selective sweep analysis. The results of LD decay were consistent with previous reports (Supplementary Data Fig. S6) [8].

**Figure 5:**
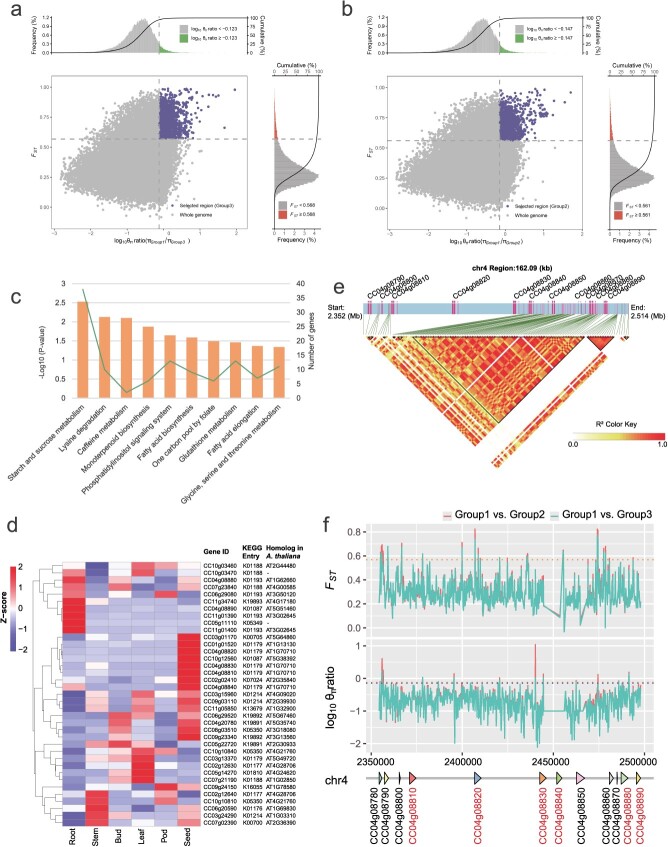
Genomic regions under selective sweep signals in *Cajanus* populations. **a** Distribution of θπ ratios (θπ, Group 1/θπ, Group 3) and *F*_ST_ values, which were calculated in 50-kb windows sliding in 5-kb steps. The blue area in the upper right corner represents the top 5% of θπ ratios (−0.123) and *F*_ST_ values (0.568), which were identified as selected regions for Group 3. **b** Distribution of θπ ratios (θπ, Group 1/θπ, Group 2) and *F_ST_* values, which were calculated in 50-kb windows sliding in 5-kb steps. The blue area in the upper right corner represents the top 5% of θπ ratios (−0.147) and *F*_ST_ values (0.561), which were identified as selected regions for Group 2. **c** KEGG pathway analysis of the genes under selective signals from Group 1 to Group 2 and Group 3. **d** Expression patterns of genes under selective signals and involved in starch and sucrose metabolism in (**c**). **e** LD block of genes involved in starch and sucrose metabolism in chr4. An inverted triangle circled by straight lines represented an LD block. **f** Example of starch and sucrose metabolism-related genes under strong selective sweep signals. θπ and *F*_ST_ values of the selected region were calculated in 100-kb windows sliding in 50-kb steps. Horizontal lines represent the top 5% tails from (**a**) and (**b**). Whole-genome resequencing data for *Cajanus* accessions were obtained from published research [8].

Furthermore, the pairwise fixation index (*F*_ST_) values between Group 1, Group 2, and Group 3 indicated a closer relationship between Group 3 and Group 2 (*F*_ST_ = 0.006) compared with the relationships between Group 3 and Group 1 (*F*_ST_ = 0.313) and between Group 2 and Group 1 (*F*_ST_ = 0.309). Additionally, we identified genomic selection regions from Group 1 to Group 2 and Group 1 to Group 3, as inferred by log_10_π ratios and *F*_ST_*.* A total of 1 666 and 1 790 potential selective sweep regions were identified from Group 1 to Group 2 and Group 1 to Group 3, respectively (Fig. 5a and b). Importantly, more than half of the selected regions (1 193 genomic regions) were shared by both evolutionary processes, comprising ~59.65 Mb or 7.33% of the assembled ‘D30’ genome. The identified selected regions encompassed 1 753 genes, which were expressed across various tissues (Supplementary Data Fig. S7). KEGG pathway enrichment analysis of these genes revealed significant involvement in ‘starch and sucrose metabolism’ (ko00500; *P* < 0.01) (Fig. 5c). Detailed functional annotation of the 38 genes involved in starch and sucrose metabolism identified six endoglucanase (K01179), four beta-glucosidase (K01188), and four beta-fructofuranosidase (K01193) genes. Among these, 16 genes were highly expressed in pigeonpea seeds (Fig. 5d). Within this subset, we analyzed LD and selection signals near the four genes on chromosome 4, identifying a 51-kb LD block containing 71 SNPs and strong selective sweep signals in Group 2 and Group 3 (Fig. 5e and f).

### Genome-wide association studies with agronomic traits of pigeonpea populations

**Figure 6:**
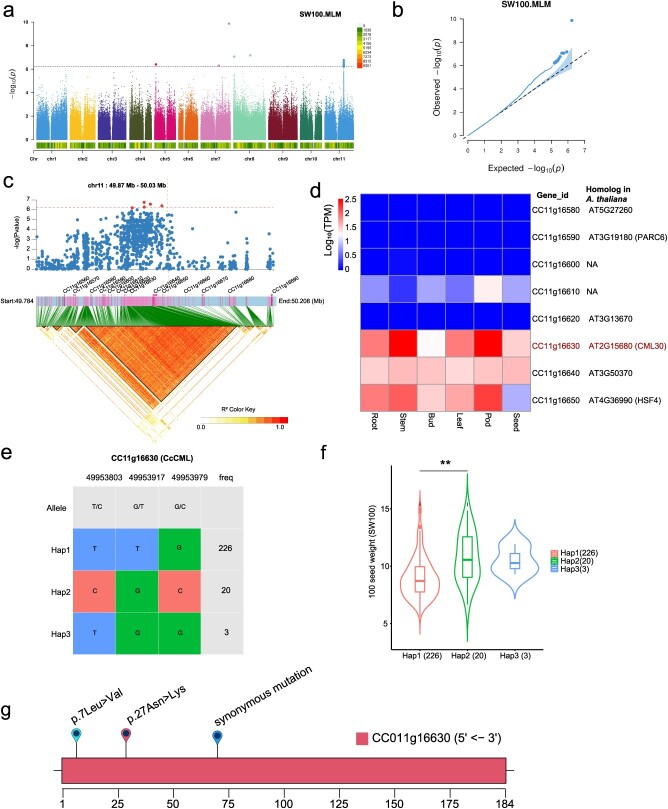
GWAS analysis of 100-seed weight (SW100) in pigeonpea. **a** Manhattan plot of GWAS results of SW100 with the MLM model. The cut line was stetted based on 1/*N*_e_, where *N*_e_ is the effective number of independent SNPs. **b** QQ plot of GWAS results of SW100 with the MLM model. **c** LD block analysis of significant association sites on chr11, which contained a 163-kb LD block and eight protein-coding genes. **d** Heat map of eight genes within the LD block shown in (**c**). **e** Haplotype analysis of calmodulin-like protein (*CcCML*, *CC11g16630*), which was highly expressed in pod, flower, and stem. **f** Haplotypic statistics of three haplotypes of the *CcCML*. There was a significant difference between Hap1 and Hap2, and Hap2 had a larger seed weight. Numbers in parentheses indicate the number of *Cajanus* accessions, and the asterisk represents *P* < 0.01. **g** Location of SNPs and the resulting amino acid changes for the *CcCML* gene. Whole-genome resequencing data and SW100 data for *Cajanus* accessions were obtained from published research [8].

To identify candidate genomic loci and genes associated with eight agronomic traits, GWAS was conducted using a mixed-model method (MLM). Biallelic SNP sites with minor allele frequencies (MAFs) of >5% were retained for the GWAS study. Data from 2 years of eight agronomic traits were retrieved from the previous report [8] and then integrated by best linear unbiased prediction (BLUP), and the extremums were removed according to the standard deviation method (3 sigma criterion). As a result, we identified a total of 28 marker–trait associations (MTAs) that were significantly associated with six agronomic traits: 11 MTAs for days to 50% flowering (DF), one MTA for primary branches per plant (PBPP), three MTAs for plant height (PH), one MTA for pods per plant (PODPP), one MTA for secondary branches per plant (SBPP), and 11 MTAs for 100 seed weight (SW100) (Fig. 6; Supplementary Data Figs S8–S14; Supplementary Data Table S17). Notably, the majority of 11 SW100-associated MTAs were concentrated on chromosome 11 (Fig. 6a). Consequently, we performed an LD analysis on chromosome 11 around the SW100-associated MTAs, revealing that these MTAs resided within a 163-kb LD block, which contained 711 SNPs and eight genes (Fig. 6c). Further gene expression analysis of these eight genes across various tissues identified a calmodulin-like protein gene (*CcCML*, *CC11g16630*) exhibiting high expression levels in both pods and seeds (Fig. 6d; Supplementary Data Table S18). Haplotype analysis of the *CcCML* gene revealed the presence of three haplotypes among the pigeonpea populations, with haplotype 2 (average SW100 of 10.71) showing a significant difference from haplotype 1 (average SW100 of 8.96) and identified as the dominant haplotype (Fig. 6e and f; Supplementary Data Table S19). Two of the three SNPs within *CcCML*, specifically at positions 49 953 917 (chr11:g.49953917 T → G) and 49 953 979 (chr11:g.49953979 G → C) on chromosome 11, were non-synonymous SNPs. These resulted in the replacement of asparagine (Asn) with lysine (Lys) and leucine (Leu) with valine (Val), respectively (Fig. 6g). Additionally, among the 11 DF-associated MTAs, 10 were concentrated on chromosome 9 (Supplementary Data Fig. S8). Furthermore, LD analysis of the genomic region around the DF-associated MTAs on chromosome 9 revealed a 29.76-kb LD block that contained two genes (*CC09g17410* and *CC09g17420*) (Supplementary Data Fig. S15). Further haplotype analyses revealed the presence of two SNPs in *CC09g17420*, which could be classified into three haplotypes. Of these, haplotype 2 was associated with early flowering (Supplementary Data Table S20, Supplementary Data Figs S16 and S17). The gene *CC09g17420* encodes a member of the ABC transporter G family (*CcABCG*) and is highly expressed in pigeonpea seeds (Supplementary Data Table S21). We further explored whether these two SNPs in *CcABCG* resulted in amino acid changes and found that the two SNPs were synonymous mutations.

## Discussion

Although pigeonpea serves as a primary protein source for resource-poor farmers in tropical and subtropical regions of developing countries, it is often classified as an orphan crop, having been domesticated by humans but not fully exploited to its potential to date [21, 22]. Genomic research on wild relatives or ancient landraces of pigeonpea will establish the foundation for genetic improvement of existing cultivars, thereby enabling the further development and utilization of pigeonpea. Here, we report a high-quality reference genome for an ancient Chinese landrace, ‘D30’, of pigeonpea. The assembled ‘D30’ genome, with a size of 813.54 Mb and contig and scaffold N50 values of 10.74 and 73.07 Mb, respectively, surpasses the contiguity achieved by the previously reported pigeonpea genome (Table 1) [10, 23, 24]. The high-quality assembled genome facilitated our further research on pigeonpea LTRs, leading to the discovery that most intact LTRs in pigeonpea have expanded recently, and that 4.80% of the LTRs reside within gene regions, thereby increasing the intron length and overall gene length of these genes (Supplementary Data Figs S2 and S3). Additionally, fragmented and incompletely assembled genomes significantly influence gene prediction [25, 26]. The number of predicted genes in various versions of the pigeonpea genome varies significantly (Table 1), possibly due to differences in the completeness of the assembled genomes. For instance, the previously reported ‘Asha’ genome was sequenced and assembled using NGS methods, resulting in fragmented sequences and a smaller assembled genome size than estimated [10]. The high-quality ‘D30’ genome generated in this study enables accurate prediction of protein-coding genes in pigeonpea. Moreover, comparing the assembled ‘D30’ genome with the published ‘Asha’ genome identified a large number of SNPs, indels, and SVs, shedding light on the diversity and adaptive traits of pigeonpea across different geographical regions (Fig. 2).

Genome resequencing of wild and cultivated accessions has served to investigate genetic variation patterns and genes that have contributed to domestication and crop improvement in soybean [27], grapevine (*Vitis vinitera*) [28], maize (*Z. mays*) [29], and rice (*Oryza sativa*) [30]. In our study, we re-analyzed the *Cajanus* accessions with resequencing data [8] leveraging the high-quality reference genome of ‘D30’. We ascertained that ‘D30’ is an ancient pigeonpea through the phylogenetic tree, PCA, and population structure analysis (Fig. 4). Additionally, we identified genomic regions under selection signals, indicating that genes enriched in starch and sucrose metabolism (ko00500; *P* < 0.01) were under selection (Fig. 5c). Among the selective genes involved in starch and sucrose metabolism, 42.11% (16 of the 38 genes) were highly expressed in the seeds of pigeonpea (Fig. 5d), suggesting that the selection of these genes might have occurred alongside the selection process for pigeonpea seeds.

A high-quality genome is crucial for the efficacy of GWAS, serving as a comprehensive and precise reference for correlating genetic variants with phenotypic traits. This fundamental step significantly improves the resolution and reliability of GWAS, enabling the accurate identification of genes associated with traits of interest [31, 32]. Previous investigations into the genetic architecture of agronomic traits in pigeonpea through GWAS have relied predominantly on second-generation assembled reference genomes and pan-genomes. However, these resources may not fully encompass the genomic intricacies [8, 11]. Based on the assembled ‘D30’ genome, our study identifies high-quality population SNPs and applies the MLM model in GWAS to identify candidate genomic loci and genes linked to eight agronomic traits reported previously [8] (Fig. 6; Supplementary Data Figs S8–S14; Supplementary Data Table S17). Previous SW100 trait association analysis based on the ‘Asha’ genome revealed scattered significant SNPs on chromosomes 4, 7, and 11 [8]. Notably, our analysis unveiled a focused peak of SNPs on chromosome 11 significantly associated with the SW100 trait, located within a 163-kb LD block encompassing a *CML* gene (*CcCML*) (Fig. 6a–c). Extensive literature underscores the critical role of *CML* genes in plant stress responses [33–36], notably highlighting the involvement of *AtCML39* in seed and fruit maturation in arabidopsis [37, 38]. Our research discovered a novel locus associated with seed weight and identified a dominant haplotype within the *CcCML* gene linked to seed weight. This finding emphasizes the potential of *CML* in regulating seed development and size, a facet that has been underexplored in pigeonpea. The observed high expression of the *CcCML* gene in reproductive tissues indicates its potential role in regulating seed phenotype (Fig. 6), corroborating findings from arabidopsis, where *CMLs* have been implicated in seed development [38]. More importantly, two non-synonymous mutant SNPs within the coding region of the *CcCML* gene are associated with the SW100 trait, leading to two amino acid changes in *CcCML* (Fig. 6g). It has been reported that missense mutations in amino acids can cause significant changes in protein function, thereby altering the associated phenotype [39–41]. Further experimental evidence is required to determine the functional impact of these mutations in *CcCML* on the SW100 trait in pigeonpea.

In this study, we also identified a consecutive peak of SNPs on chromosome 9 that exhibited a significant association with the DF trait (Supplementary Data Fig. S8), consistent with findings from previous GWAS studies on the DF trait in pigeonpea [8]. Furthermore, a particular haplotype of *CcABCG* (*CC09g17420*) is significantly associated with early flowering in pigeonpea (Supplementary Data Figs S16 and S17). Studies on the model plant arabidopsis have demonstrated that multiple ABCG members are involved in the transport of plant hormones, such as abscisic acid, cytokinin, and the auxin precursor IBA (indole-3-butyric acid) [42–46]. The *CcABCG* gene in pigeonpea could potentially affect flowering time through the translocation of plant hormones. However, two SNPs within the coding region of the *CcABCG* gene were synonymous mutations, meaning these two SNPs did not alter any amino acids. It has been reported that synonymous mutations may impact gene expression and function through changes in post-transcriptional processing and RNA regulation, modifying both local and global mRNA structures, and influencing translation kinetics [47–50]. Thus, further investigation is needed to understand how SNP alterations in the *CcABCG* gene affect the flowering time of pigeonpea.

### Conclusions

Our study has successfully generated a high-quality, chromosome-scale reference genome for the ancient Chinese landrace ‘D30’ of pigeonpea. The comprehensive genome annotation, comparative genomic analysis, and insights into population variations significantly enhance our understanding of pigeonpea genetics and evolution. These findings contribute to advancing research on pigeonpea, emphasizing the critical role of genomic resources in addressing global food security challenges.

## Materials and methods

### Plant material

The pigeonpea germplasm ‘D30’ used in this study was provided by the Tropical Crops Genetic Resources Institute (TCGRI), Chinese Academy of Tropical Agricultural Sciences (CATAS), Hainan, China.

### Genomic sequencing

Young leaves of ‘D30’ were selected for DNA extraction. High-quality extracted DNA was used for HiFi library and NGS library construction. PacBio HiFi reads were sequenced on the PacBio Sequel II system (HiFi mode) and NGS data were sequenced on the MGISEQ-2000 sequencing platform by BGI Tech (Shenzhen, China). Young and tender leaves of ‘D30’ were collected for Hi-C sequencing on the MGISEQ-2000 sequencing platform by BGI Tech (Shenzhen, China). Roots, stems, buds, leaves, flowers, seeds, and pods of ‘D30’ were collected for transcriptome sequencing on the MGISEQ-2000 sequencing platform by BGI Tech (Shenzhen, China). Raw data of NGS, Hi-C, and RNA-seq were subjected to quality control and filtering analysis using SOAPnuke (version 2.1.6) [51]. Detailed information is provided in supplementary information: Supplementary Data Method 1.

### Genome assembly and annotation

CCS reads of the genome of ‘D30’ were assembled by applying hifiasm (version 0.14.2-r315) [52]. Hi-C reads were mapped onto the assembled contigs using Burrows–Wheeler Aligner (BWA, version 0.7.17), and juicer (https://github.com/theaidenlab/juicer) was adopted for annotation of Hi-C map features. Additionally, the 3D-DNA (https://github.com/aidenlab/3d-dna) pipeline was employed to assemble contigs into chromosomal pseudomolecules. BUSCO (version 5.1.0) [53] and LAI analysis were adopted to assess the assembly quality of the assembled ‘D30’ genome. NGS and PacBio HiFi reads were mapped onto the ‘D30’ genome using BWA (version 0.7.17) and winnowmap (version 2.03), respectively, to calculate the genome alignment rate. The pigeonpea genome annotation includes the annotation of repetitive sequences, protein-coding genes, and non-coding RNAs. We adopted both *de novo* prediction and homology-based prediction methods for repetitive sequence identification. LTR_Finder (version 1.0.7), LTR_retriever (version 1.9), RepeatModeler (version 2.0.1), and RepeatScout (version 1.0.6) were employed for *de novo* prediction of repetitive sequences. The known repetitive sequence database RepBase (version 20120418) was adopted for homology-based prediction. Protein-coding genes were predicted based on evidence from *de novo*, homology-based prediction, and transcriptomic-based prediction. EVidenceModeler (https://github.com/EVidenceModeler/EVidenceModeler) was employed to integrate all predicted evidence to obtain the final non-redundant set of protein-coding genes. We used BLAST (version 2.2.23) to align protein sequences to known databases, including the NCBI Non-Redundant Protein Sequence Database (NR), KEGG, Eukaryotic Orthologous Groups of Protein (KOG), Swiss-Prot, and TrEMBL, for functional annotation of protein-coding genes. Based on the NR annotation, we adopted Blast2GO (version 6.0) for GO annotation. Additionally, InterProScan (version 5.59–91.0) was adopted to identify protein structural domains, and DRAGO 2 (http://prgdb.org/prgdb/drago2) was adopted to annotate resistance genes. Detailed information is provided in supplementary information: Supplementary Data Method 1.

### Comparative genomic analysis

Genetic variants (including SNPs, indels, and SVs) between ‘Asha’ and ‘D30’ were conducted using NGS and third-generation sequencing (TGS) data. NGS data of ‘Asha’ (SRR5922906) and TGS data of ‘Asha’ (SRR10053121) were aligned to the ‘D30’ genome using BWA (version 0.7.17) and ngmlr (version 0.2.7), respectively. SNPs and indels were identified using GATK (version 4.1.2.0) based on the NGS mapping results. SVs were identified using sniffles (version 1.0.11), cuteSV (version 2.1.1), pbsv (version 2.9.0), and SVIM (version 2.0.0) based on the TGS mapping results. SVs supported by at least two methods were retained for subsequent analyses. SnpEff (version 5.1) was employed for SNP and indel annotation, and BEDTools (version 2.30.0) was employed to identify genomic regions and genes affected by SVs. SNPs, indels, and SVs that resided in exon regions of genes were considered as high-impact variants. OrthoMCL (version 2.0.9) was adopted for OG identification among the studied species. Further gene family expansion and contraction analysis was conducted using CAFÉ (version 2.1). Based on single-copy gene families, multiple sequence alignment was performed using MUSCLE (version 3.8.31). Phylogenetic trees of the studied species were constructed using PhyML (version 3.0). Furthermore, MCScanX (https://github.com/wyp1125/MCScanX) was employed for intra- and inter-species gene collinearity analysis, and duplicate_gene_classifier was adopted for classifying duplicated genes based on our previously reported strategy [54]. The synonymous (*K*_s_) and non-synonymous (*K*_a_) substitution rates of gene pairs in collinear regions were calculated by employing PAML (version 4.9e) and PAL2NAL (version 14) using the Nei–Gojobori (NG) method [55]. The formula *T* = *K*_s_/2*r* was used to calculate the divergence time, where the neutral substitution rate *r* was selected as 6.1 × 10^−9^  *K*_s_/year [56–58]. Variant-affected genes were classified into cellular component (CC), molecular function (MF), and biological process (BP) based on the GO annotation terms. KEGG pathway enrichment analysis of the ‘D30’ genome expanded genes was performed using *phyper* and *p.adjust* functions under the R platform (version 4.2.0), and GO terms and KEGG pathway with FDR < 0.05 were considered as significantly enriched. Detailed information is provided in supplementary information: Supplementary Data Method 2.

### Population genetics analysis

DNA resequencing data of 292 *Cajanus* accession (BioProject: PRJNA383013) and ‘Asha’ (SRA run: SRR5922906) were retrieved from the SRA database (https://www.ncbi.nlm.nih.gov/sra/). Quality control of the sequencing data was performed using SOAPnuke (version 2.1.6) [51]. Clean reads of each *Cajanus* accession were mapped to the ‘D30’ genome using BWA (version 0.7.17), with bam sorting and PCR duplicate removal handled by SAMtools (version 1.7) and Picard (version 2.18.16), respectively. Variants from each accession were detected by GATK (version 4.1.2.0) and the genomic variant call format (GVCF) of all *Cajanus* accessions were combined by CombineGVCFs. Population variations were filtered by VariantFiltration. VCFtools (version 0.1.16) was adopted for filtering of the max-missing ratio (0.5) and minor allele frequency (MAF, 0.05). Population structure analyses were performed using admixture (version 1.3.0) by incrementally varying *K* (the number of populations) from 2 to 10 and assessing the CV error [59, 60]. Since none of the *K* values from 2 to 10 exhibited minimal CV error, and given that the population phylogenetic tree distinctly clustered into seven groups, we visualized the results of the population structure analysis using pophelper (version 2.3.1) for *K* values ranging from 2 to 7. PCA was performed using GCTA (version 1.94.1). Population *p*-distance matrix and phylogenetic tree analyses were performed using VCF2Dis (version 1.47) and PHYLIPNEW (version 3.69.650), respectively.

### Genome-wide selective sweep analysis

Based on the analyses of population evolution, PCA, and population structure, we designated six wild varieties and four landraces (including ‘D30’ in this study) as Group 1. This group included 10 *Cajanus* accessions which represent the wild and ancient pigeonpea. The landraces not included in Group 1 were designated as Group 2, and the breeding lines were designated as Group 3. We searched for pigeonpea whole-genome selective sweep signals based on nucleotide diversity (π) and the fixation index (*F*_ST_). The analyses of π and *F*_ST_ for Group 1, Group 2, and Group 3 were performed using VCFtools (v0.1.17), adopting a window size of 50 000 and a step size of 5 000. Windows with the top 5% of π and *F*_ST_ values were considered as selection regions, i.e. genomic regions with log_10_θ_π_ ≥ −0.123 and *F*_ST_ ≥ 0.568 for Group 3 versus Group 1, and log_10_θ_π_ ≥ −0.147 and *F*_ST_ ≥ 0.561 for Group 2 versus Group 1, were considered as genomic selection regions. Genes within these genomic selection regions were considered as candidate selective genes. KEGG pathway enrichment analysis of these selected genes was performed under the R platform (version 4.2.0) using a hypergeometric test.

### GWAS analyses

In our study, eight agronomic traits collected over two years were retrieved from a previous report [8]. These traits were integrated by the BLUP method, and the extremes were removed according to the standard deviation method (3 sigma criterion). The MLM method is highly preferred in GWAS because it effectively corrects inflation caused by numerous small genetic effects (polygenic background) and addresses bias from population stratification [61–66]. In this study, GWAS analysis for each trait was performed by rMVP (https://github.com/xiaolei-lab/rMVP) [67] with the MLM model, and cmplot (version 4.4.1) was applied to draw QQ and Manhattan plots for each trait. The Bonferroni correction (1/*N*_e_) (6.21E−07 in this study) was used as a cutoff line on the Manhattan plot, in which *N*_e_ was the effective number of independent SNPs calculated by the Genetic Type I Error Calculator (GEC), which has been implemented in KGGSEE (version 1.1) [68]. SNPs with a *P* value below this threshold were considered as MTAs. LD heat maps of the region of interest were conducted by LDBlockShow (https://github.com/BGI-shenzhen/LDBlockShow) based on the *R*^2^ statistic. Heat maps of gene expression were generated by pheatmap (version 1.0.12). Associations between SNP loci in the *CcCML* gene and the SW100 trait, as well as the *CcABCG* gene and the DF trait, were analyzed using the Shapiro–Wilk and ANOVA tests under the R platform (version 4.2.0). Haplotype analysis of the *CcCML* and *CcABCG* gene was performed by geneHapR (https://github.com/ZhangRenL/geneHapR).

## Supplementary Material

Web_Material_uhae201

## Data Availability

The raw genomic sequencing data, including PacBio HiFi, BGISEQ, Hi-C, and transcriptome sequencing data, are available in the National Genomics Data Center (NGDC) under PRJCA024778. DNA resequencing data of 292 *Cajanus* accessions (BioProject: PRJNA383013), NGS data (SRR5922906), and TGS data (SRR10053121) of ‘Asha’ were retrieved from the SRA database (https://www.ncbi.nlm.nih.gov/sra/). The genome assembly and gene annotation reported in this paper have been deposited in the Genome Warehouse in National Genomics Data Center, Beijing Institute of Genomics (China National Center for Bioinformation), Chinese Academy of Sciences, under accession number GWHETRU00000000.1, which is publicly accessible at https://bigd.big.ac.cn/gwh.
