# Supplementary material for: Chromosome-scale reference genome of an ancient landrace: unveiling the genetic basis of seed weight in the food legume crop pigeonpea (Cajanus cajan)
Source: Hortic Res. 2024 Jul 30;11(9):uhae201. doi: 10.1093/hr/uhae201 (PMC11387010; doi:10.1093/hr/uhae201)
Supplement: Web_Material_uhae201 [file web_material_uhae201.zip › Figure S16.pdf]

CC09g17420 (ABCG)

	55919547	55919664	freq
Allele	T/C	G/A	
Hap1	C	G	236
Hap2	T	A	8
Hap3	T	G	3
