# Supplementary material for: Chromosome-scale reference genome of an ancient landrace: unveiling the genetic basis of seed weight in the food legume crop pigeonpea (Cajanus cajan)
Source: Hortic Res. 2024 Jul 30;11(9):uhae201. doi: 10.1093/hr/uhae201 (PMC11387010; doi:10.1093/hr/uhae201)
Supplement: Web_Material_uhae201 [file web_material_uhae201.zip › Supplementary information.docx]

**Chromosome-scale reference genome of an ancient landrace: unveiling the genetic basis of seed weight in the** **food legume crop pigeonpea (*Cajanus cajan*)**

Chun Liu^1,2,3,4,^ ^†^, Xipeng Ding^1,^ ^†^, Yuanhang Wu^5^, Jianyu Zhang^4^, Rui Huang^1^, Xinyong Li^1^, Guodao Liu^1,^*, and Pandao Liu^1,2,3,^*

# Supplementary Method 1. Genomics sequencing and assembly

## Library construction and sequencing

Young leaves of D30 were selected for DNA extraction. DNA concentration was measured using a Qubit Fluorometer and NanoDrop, and the integrity of the samples was assessed with a 1% agarose gel. The results showed a sample concentration of 85.8 ng/μL, a total amount of 17.16 μg, an OD260/280 of 1.99, and an OD260/230 of 2.05. The extracted DNA was of high quality and met the requirements for further experiments. The same sample was divided into two portions: one for high-fidelity (HiFi) library construction and the other for second-generation sequencing library construction.

For HiFi sequencing, quality-checked DNA samples were sheared using Megaruptor, followed by the selection of 13–16 kb DNA fragments using SageELF. Subsequently, the fragments were blunted, and circular single-stranded adaptors were ligated to both ends, forming a structure similar to SMRT Bell "lasso" rings. This process, involving precise shearing by Megaruptor and accurate selection and recovery by SageELF, resulted in a DNA library with very high consistency in fragment size for sequencing on the PacBio Sequel II system (HiFi mode). During the PacBio sequencing process, each DNA fragment's double strands are circularly sequenced multiple times. High-quality, long-read sequencing results (CCS sequences) are obtained using smrtlink (version 8.0).

For second-generation sequencing (NGS), a Quality-checked sample is sheared ultrasonically using a Covaris instrument, followed by DNA fragment selection, end repair, and adapter ligation. The MGISEQ-2000 sequencing platform is used for read lengths of 150 bp in paired-end high-throughput sequencing. The raw data from the sequencing machine are subjected to quality control and filtering analysis using SOAPnuke (version 2.1.6)^1^, resulting in high-quality clean data.

The young and tender leaves of D30 are collected for Hi-C sequencing. Then use formaldehyde to fix the natural state of DNA-protein or protein-protein complexes within cells. Chromatin is digested and separated with the restriction enzyme DpnII, while the ends of the fragments are labeled with biotin. Use DNA ligase to connect the ends to form circular chimeric molecules, then after purification, cutting, and capturing, select the appropriate size DNA fragments to construct the Hi-C library. The MGISEQ-2000 sequencing platform is used for paired-end high-throughput sequencing of the constructed Hi-C library. Raw data are subjected to quality control and filtering analysis using SOAPnuke (version 2.1.6)^1^.

For transcriptome sequencing, separately extract the roots, stems, buds, leaves, flowers, seeds, and pods of D30, and use the Agilent 2100 Bioanalyzer to check the quality of the samples. Enrich mRNA from total RNA samples using oligo (dT) magnetic beads, and fragment the mRNA with fragmentation reagents. Synthesize single-stranded cDNA and double-stranded cDNA on a PCR instrument, followed by end repair of the double-stranded cDNA. Next, add an A base and adapter sequences to the 3' end. Then, in the PCR reaction system, amplify and circularize the ligation products. After digesting un-circularized linear DNA and analyzing, obtain single-stranded circular products, which constitute the transcriptome library. After preparing the transcriptome library, perform high-throughput sequencing on the MGI2000 sequencing platform with a read length of 150 bp. Use the SOAPnuke (version 2.1.6)^1^ software for quality control and filtering of the raw data.

## Genome assembly and annotation

First, high-quality NGS data of D30 were used for genome survey analysis. Jellyfish (https://github.com/gmarcais/Jellyfish) was employed to count the 21-mer sequences and frequencies of the pigeonpea NGS data. GenomeScope (<http://qb.cshl.edu/genomescope/>) was then used to plot the k-mer frequency distribution and estimate genome size and heterozygosity. The genome of D30 was assembled using CCS from Pacbio HiFi sequencing by applying Hifiasm (version 0.14.2-r315)^2^. The initial assembled contigs were utilized Hi-C to assist in the chromosome assembly by employing juicer (https://github.com/theaidenlab/juicer) and 3D-DNA (https://github.com/aidenlab/3d-dna). Chromosome-level genomes assembled with Hi-C support are divided into 100 kb bins, and the number of Hi-C reads covering any two bins was used as the interaction intensity signal between them. By plotting a heatmap of signal intensity, we can intuitively evaluate the effectiveness of Hi-C-assisted chromosome assembly. To assess the assembly quality of the assembled D30 genome, we first used BUSCO (version 5.1.0)^3^, hmmsearch (version 3.1), and the embryophyta database (embryophyta_odb10) for the assessment of conserved gene completeness. The LTR Assembly Index (LAI) analysis was conducted using LTR_retriever (version 1.9)^4^. NGS and Pacbio HiFi reads were mapped onto the D30 genome using BWA (version 0.7.17) and winnowmap (version 2.03), respectively, to calculate the genome alignment rate.

The annotation of the pigeonpea genome includes annotation of repetitive sequences, protein-coding genes, and non-coding RNAs. For repetitive sequence annotation, analyses were conducted using both *De novo* prediction and homology-based prediction methods. *De novo* annotation tools include LTR_Finder (version 1.0.7), LTR_retriever (version 1.9), RepeatModeler (version 2.0.1), and RepeatScout (version 1.0.6). Repetitive sequences predicted by these tools were integrated into a *De novo* database, and *De novo* prediction of repetitive sequences in the pigeonpea genome was performed using RepeatMasker (version 4.0.7). Homology prediction was performed based on the known repetitive sequence database RepBase (version 20120418), using RepeatMasker (version 4.0.7) and RepeatProteinMask (version 4.0.7) for homology-based repetitive sequence prediction. Additionally, tandem repeat sequences were predicted using TRF (version 4.09). The repetitive sequence results obtained from the above methods were integrated to create a dataset of repetitive sequences in the pigeonpea genome, and the repetitive sequences were classified and statistically analyzed.

Protein-coding genes were predicted based on *De novo*, homology-based predictions, and transcriptomic evidence. SNAP (version 2006-07-28) and AUGUSTUS (version 3.3.1) software were used for ab initio gene prediction. Homology-based annotation employed species such as soybean (*G. max*), Arabidopsis, white lupin (*Lupinus albus*), barrel medic (*M. truncatula*), common bean (*P. vulgaris*), grape (*Vitis vinifera*), and the published pigeonpea genome Asha for homology-based prediction, using tBLASTn (version 2.2.23) and GeneWise (version 2.4.1) for gene structure prediction based on homology. Transcriptome data from different tissues were first aligned to the pigeonpea genome using HISAT2 (version 2.1.0), followed by transcript reconstruction with StringTie (version 2.0). Finally, EVidenceModeler (https://github.com/EVidenceModeler/EVidenceModeler) was adopted for gene set integration to obtain the final non-redundant set of protein-coding genes. The completeness of the predicted gene set was analyzed using BUSCO (version 5.1.0) and the embryophyta database (embryophyta_odb10).

Non-coding RNA (ncRNA) prediction was conducted using homology-based annotation methods. Based on the Rfam (version 12.0) database, BLASTn (version 2.2.23) software and INFERNAL (version 1.0) were used for annotation of small RNAs (miRNA) and small nuclear RNAs (snRNA) in the mung bean genome. tRNAscan-SE (version 1.3.1) software was used for annotation of transfer RNAs (tRNA) in the mung bean genome. Based on a known database of plant ribosomal RNAs (rRNA), BLASTn (version 2.2.23) software was used for rRNA annotation in the pigeonpea genome.

# Supplementary Method 2. Comparative genomic analysis

Genetic variants (including SNPs, indels, and SVs) between Asha and D30 were conducted using NGS and third-generation sequencing (TGS) data. Initially, NGS data of Asha (SRR5922906) were aligned to the D30 genome using BWA (version 0.7.17), with PCR duplicates and sorting of the alignment results being handled using SAMtools (version 1.7) and Picard (version 2.18.16). Subsequently, variant detection and filtering were performed using GATK (version 4.1.2.0), extracting SNP and indel information. TGS data of Asha (SRR10053121) were aligned to theD30 genome using ngmlr (version 0.2.7) and SVs were detected using sniffles (https://github.com/fritzsedlazeck/Sniffles, version 1.0.11), cuteSV (<https://github.com/tjiangHIT/cuteSV>, version 2.1.1), pbsv (<https://github.com/PacificBiosciences/pbsv>, version 2.9.0), and SVIM (version 2.0.0) based on the TGS mapping results, and SVs supported by at least two methods were retained for subsequent analyses.

We utilized OrthoMCL (version 2.0.9) to identify gene families in pigeonpea (including D30 and Asha), Soybean, common bean, green bean (*Vigna radiata*), cowpea (*Vigna unguiculata*), chickpea (*Cicer arietinum*), white lupin, two wild species of cultivated peanut (*A. duranensis* and *A. ipaensis*), barrel medic (*M. truncatula*), and Arabidopsis (*A. thaliana*). For genes with multiple transcripts, the longest transcript was chosen to represent the gene. Initially, protein sequences were compared using BLASTp (version 2.2.23, e-value set to 1e^-5^) in an all-vs-all alignment, followed by clustering of the comparison results using OrthoMCL (version 2.0.9) with the settings “percentMatchCutoff=50, evalueExponentCutoff=-5, --abc -I 1.5,” to obtain gene family information. The clustering results were analyzed to identify single-copy families, multi-copy gene families, and species-specific gene families for further gene family expansion and contraction analysis using CAFÉ (version 2.1). Based on single-copy gene families, multiple sequence alignment was performed using MUSCLE (version 3.8.31). finally, phylogenetic trees were constructed using PhyML (version 3.0). Divergence times between chickpea and barrel medic, two wild species of cultivated peanut (*A. duranensis* and *A. ipaensis*), as well as soybean and common bean, were queried on timetree (http://www.timetree.org/) as known species divergence times. The substitution rates were then estimated using the MCMCTREE program within the PAML (version 4.5) software package, further calculating the divergence times between species.

Our study employed MCScanX (https://github.com/wyp1125/MCScanX) for intra- and inter-species gene collinearity analysis. Initially, protein sequences were compared using BLASTp (version 2.2.23, e-value set to 1e^-5^), followed by MCScanX to identify collinear regions. For intra-species collinearity results, the duplicate_gene_classifier within MCScanX classified paralogous genes into single-copy genes, dispersed duplicated genes, proximal duplicated genes, tandem duplicated genes, and whole genome or segmental duplicated genes. The synonymous (Ks) and nonsynonymous (Ka) substitution rates of gene pairs in collinear regions were calculated using PAML (version 4.9e) and PAL2NAL (version 14), obtaining the results of the Ka and Ks using the Nei-Gojobori (NG) method^5^. Ks distribution graphs were plotted using R (version 4.0.2), and Ks peaks were identified. The formula T=Ks/2r was used to calculate the divergence time, where the neutral substitution rate r was selected as 6.1×10^-9^ Ks yr^-16–8^.

1 Chen Y, Chen Y, Shi C. *et al.* SOAPnuke: A MapReduce acceleration-supported software for integrated quality control and preprocessing of high-throughput sequencing data. *Gigascience* 2018; **7**. doi:10.1093/gigascience/gix120.

2 Cheng H, Concepcion GT, Feng X. *et al*. Haplotype-resolved de novo assembly using phased assembly graphs with hifiasm. *Nat Methods* 2021; **18**. doi:10.1038/s41592-020-01056-5.

3 Simão FA, Waterhouse RM, Ioannidis P. *et al*. BUSCO: Assessing genome assembly and annotation completeness with single-copy orthologs. *Bioinformatics* 2015; **31**. doi:10.1093/bioinformatics/btv351.

4 Ou S, Jiang N. LTR_retriever: A highly accurate and sensitive program for identification of long terminal repeat retrotransposons. *Plant Physiol* 2018; **176**. doi:10.1104/pp.17.01310.

5 Nei M, Gojobori T. Simple methods for estimating the numbers of synonymous and nonsynonymous nucleotide substitutions. *Mol Biol Evol* 1986; **3**. doi:10.1093/oxfordjournals.molbev.a040410.

6 Van K, Kim DH, Cai CM. *et al.* Sequence level analysis of recently duplicated regions in soybean [Glycine max (L.) Merr.] genome. *DNA Research* 2008; **15**. doi:10.1093/dnares/dsm035.

7 Schlueter JA, Dixon P, Granger C. *et al.* Mining EST databases to resolve evolutionary events in major crop species. *Genome* 2004; **47**. doi:10.1139/G04-047.

8 Lynch M, Conery JS. The evolutionary fate and consequences of duplicate genes. *Science (1979)* 2000; **290**. doi:10.1126/science.290.5494.1151.
