# Supplementary figures and images for: Chromosome-scale reference genome of an ancient landrace: unveiling the genetic basis of seed weight in the food legume crop pigeonpea (Cajanus cajan)
Source: Hortic Res. 2024 Jul 30;11(9):uhae201. doi: 10.1093/hr/uhae201 (PMC11387010; doi:10.1093/hr/uhae201)

# GenomeScope Profile

len:823,552,273bp uniq:41.7% het:0.238% kcov:18.3 err:0.776% dup:1.09% k:21

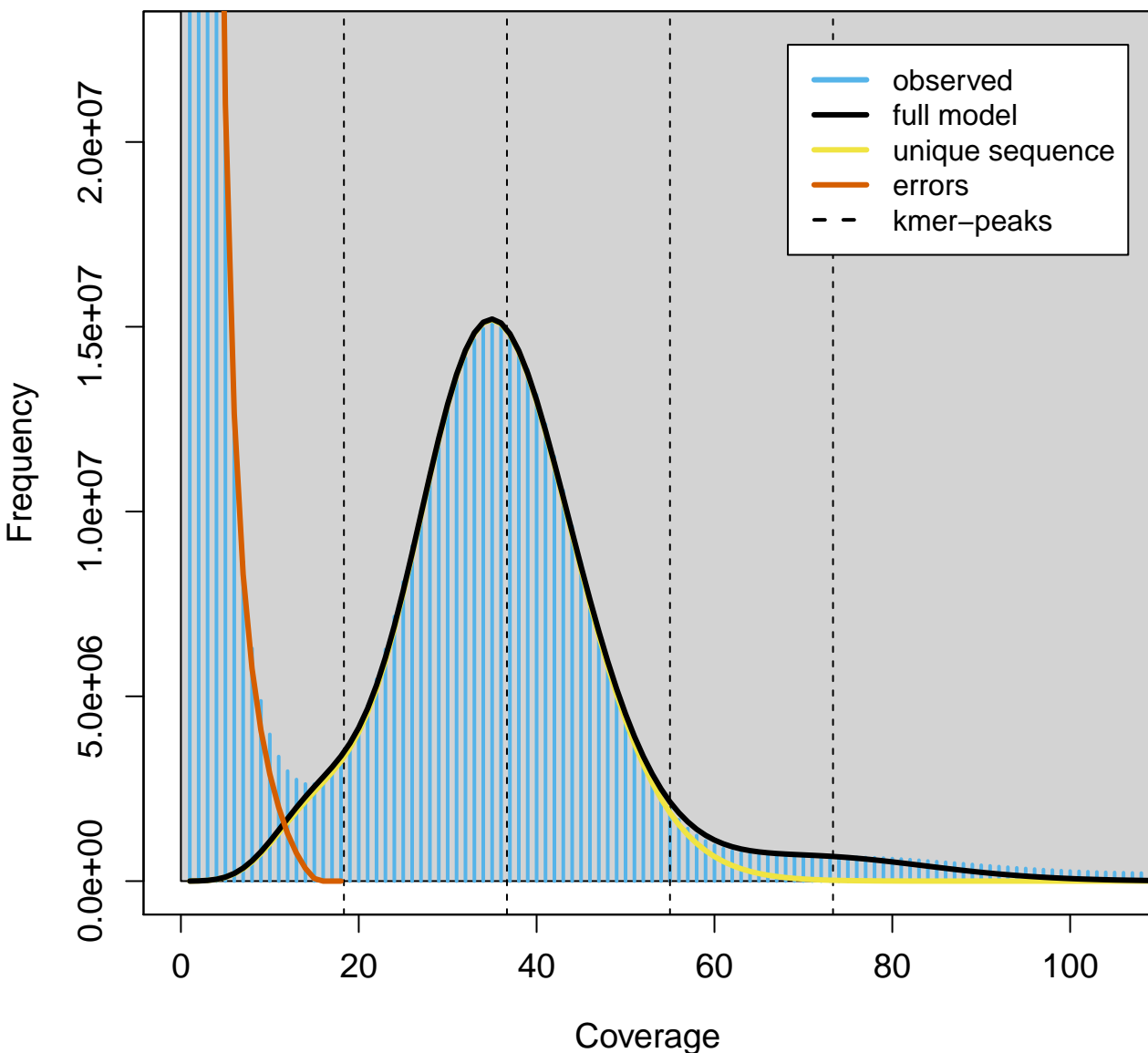

Supplement: Web_Material_uhae201 [file web_material_uhae201.zip › Figure S1.pdf]

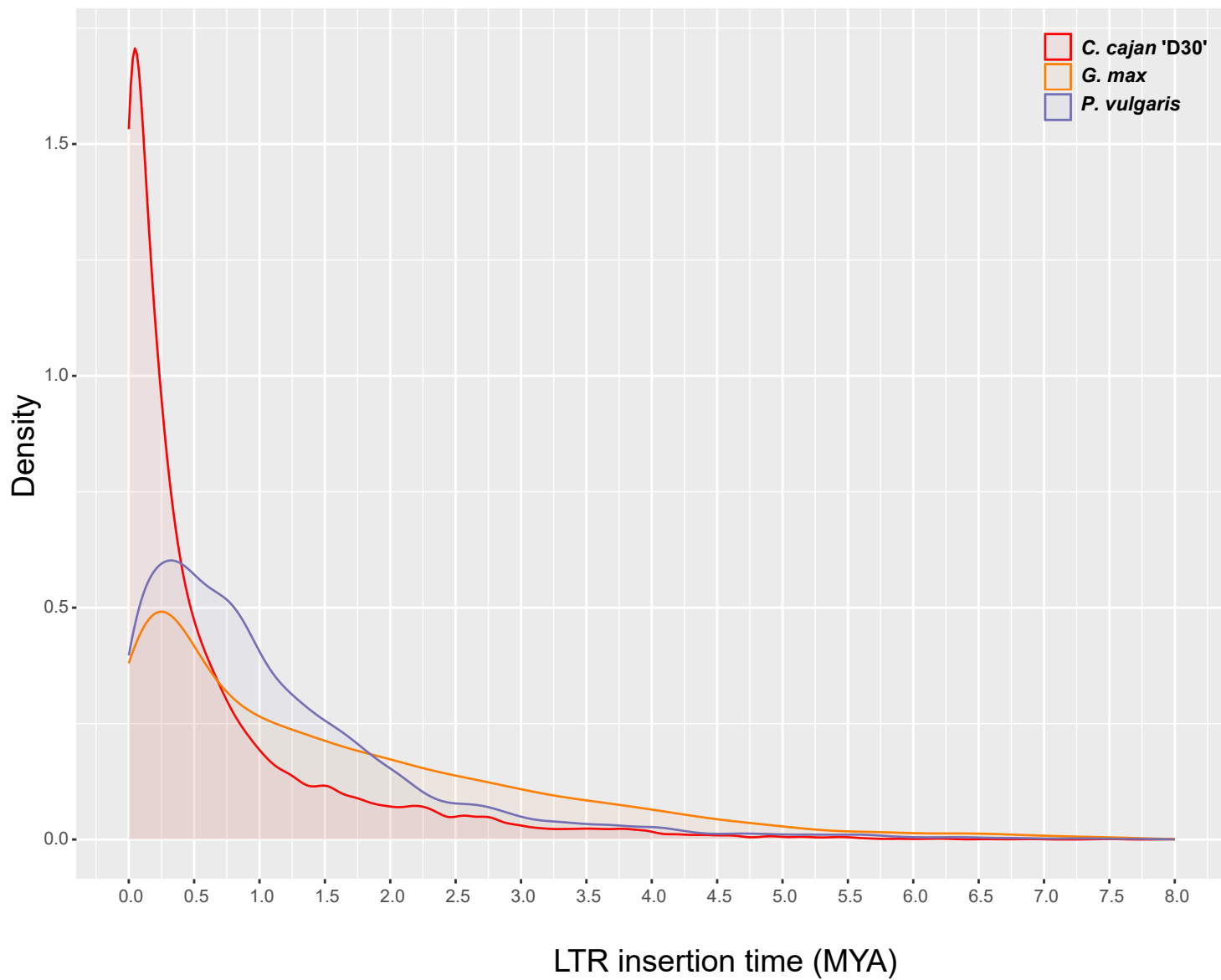

Supplement: Web_Material_uhae201 [file web_material_uhae201.zip › Figure S2.pdf]

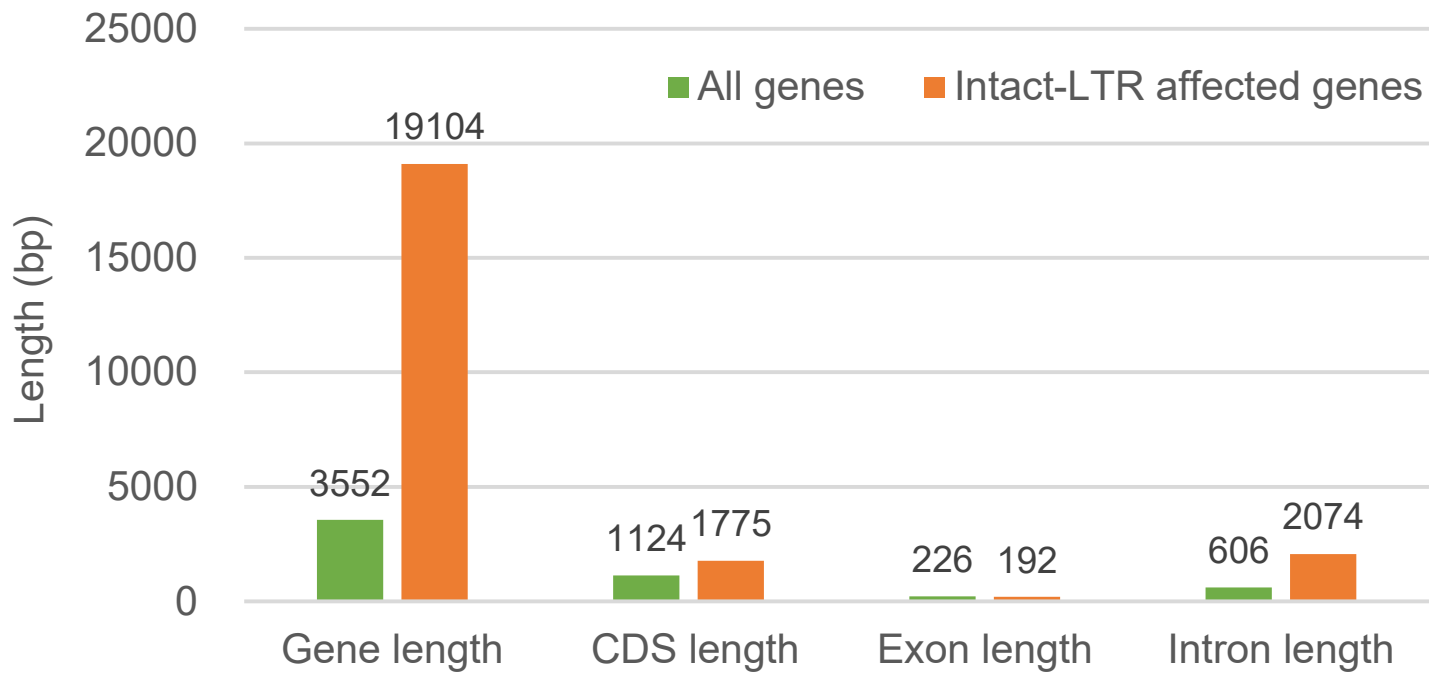

Supplement: Web_Material_uhae201 [file web_material_uhae201.zip › Figure S3.pdf]

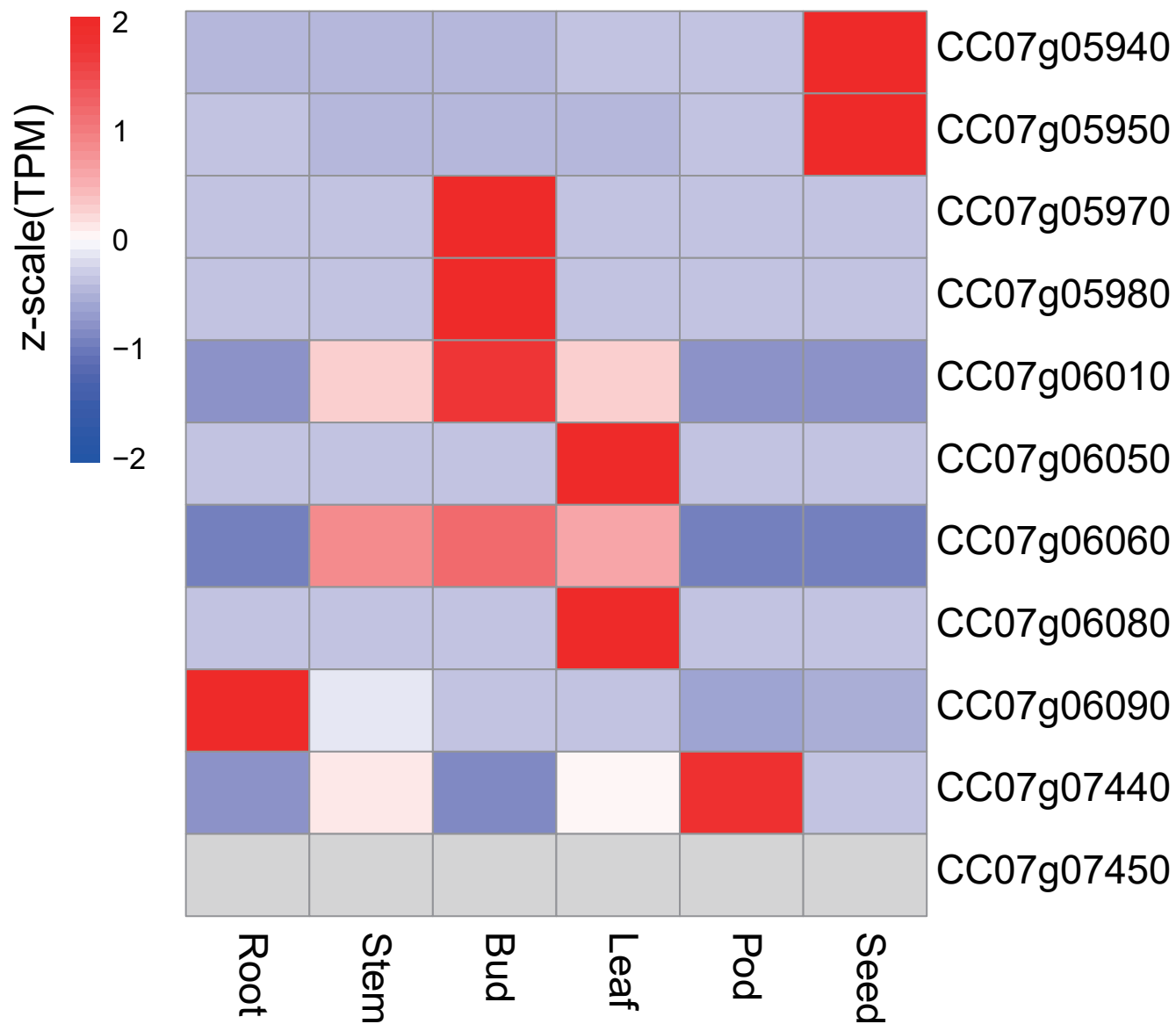

Supplement: Web_Material_uhae201 [file web_material_uhae201.zip › Figure S4.pdf]

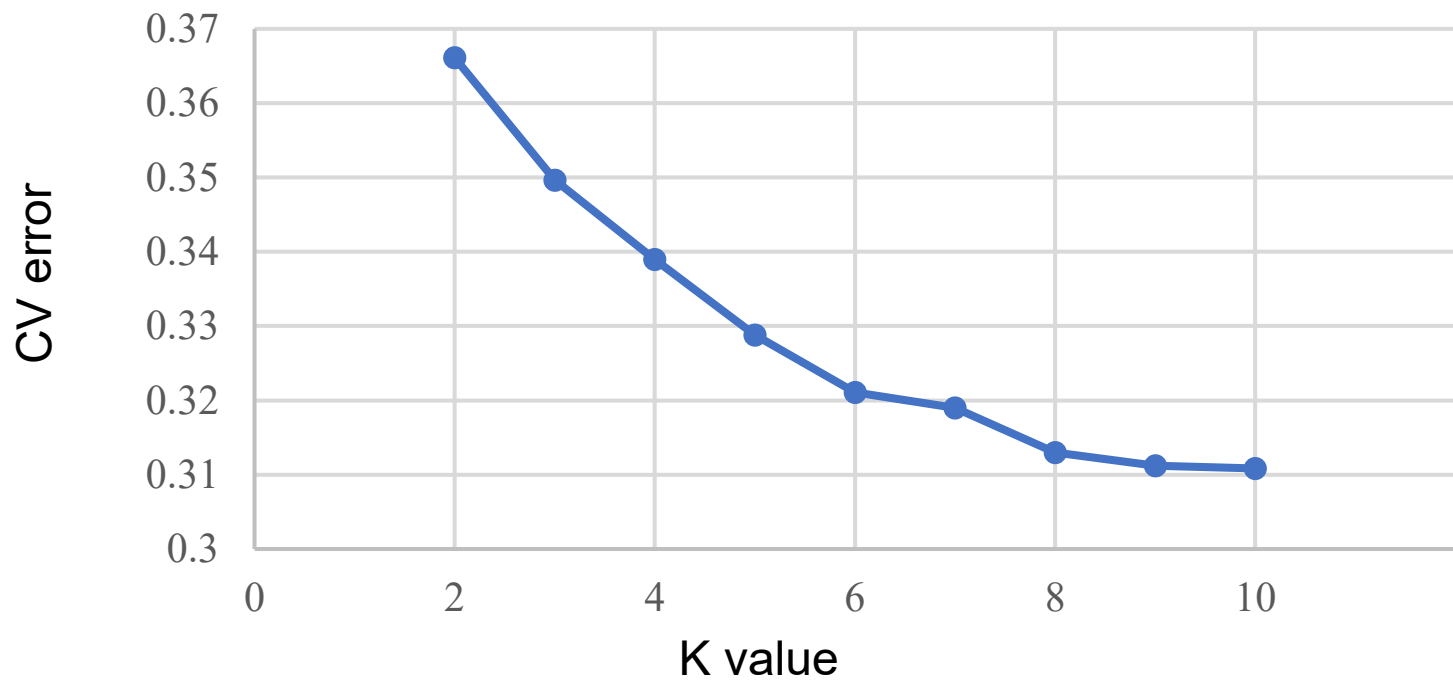

Supplement: Web_Material_uhae201 [file web_material_uhae201.zip › Figure S5.pdf]

## LD decay

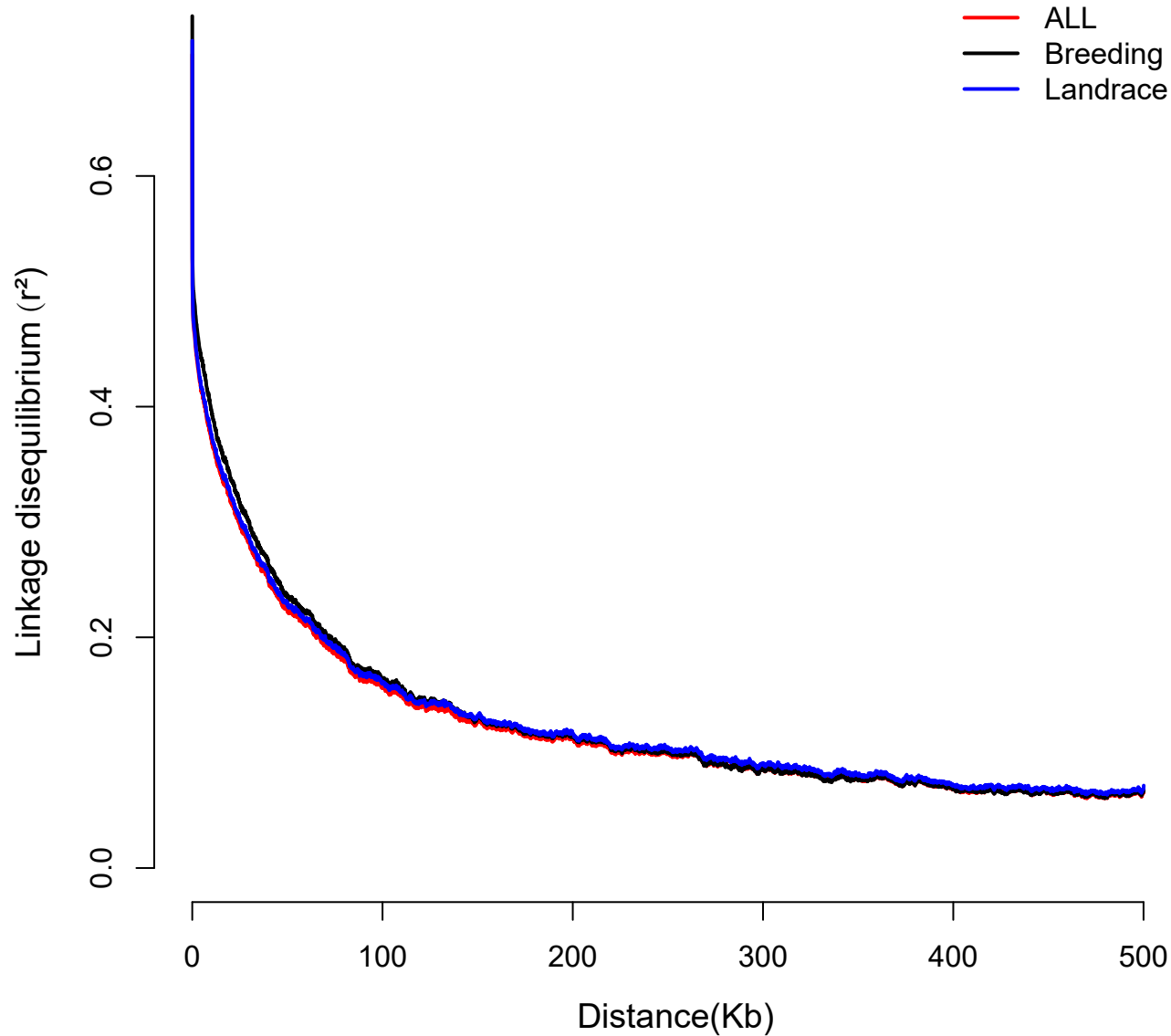

Supplement: Web_Material_uhae201 [file web_material_uhae201.zip › Figure S6.pdf]

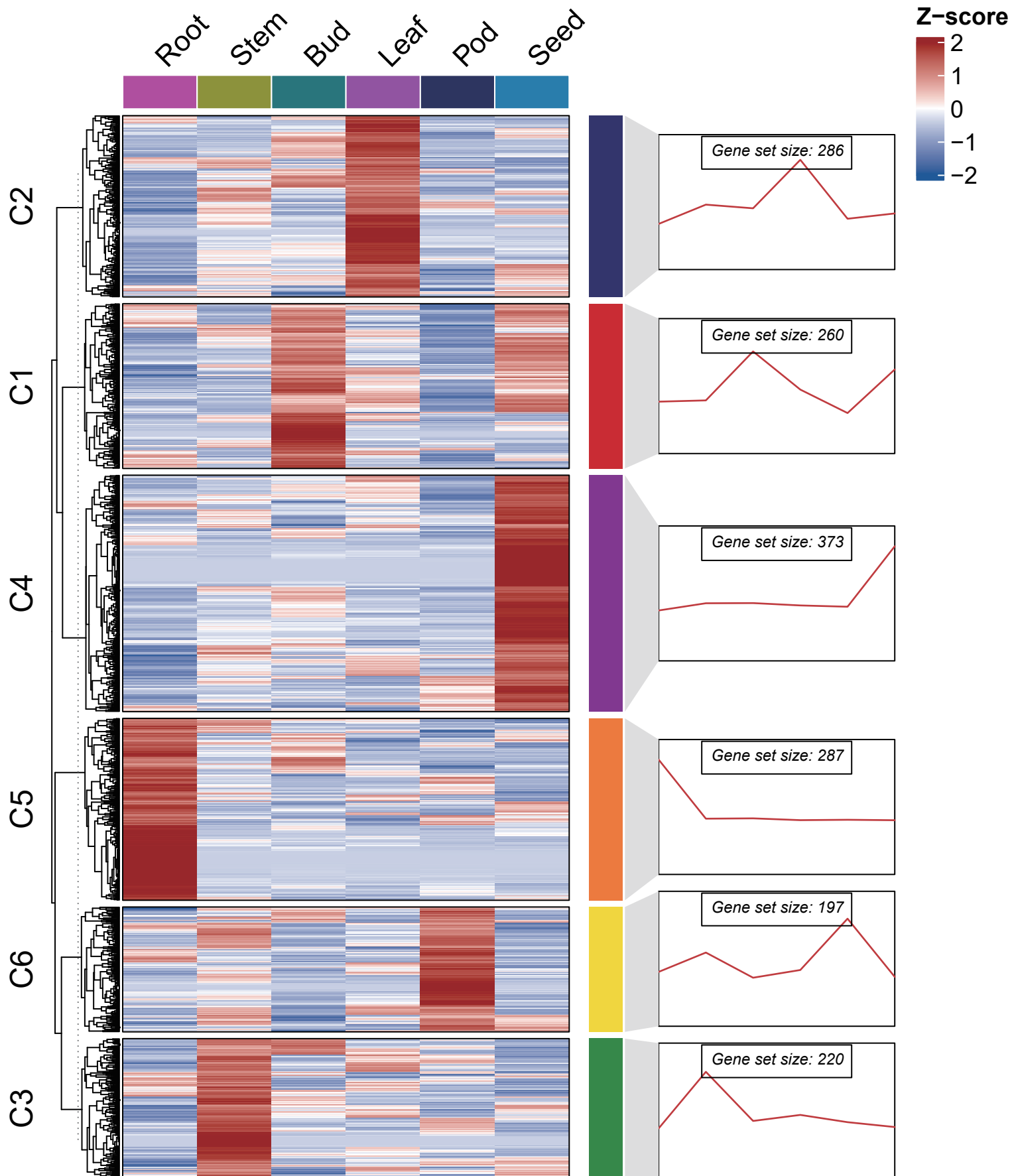

Supplement: Web_Material_uhae201 [file web_material_uhae201.zip › Figure S7.pdf]

## Days to 50% flowering (DF)

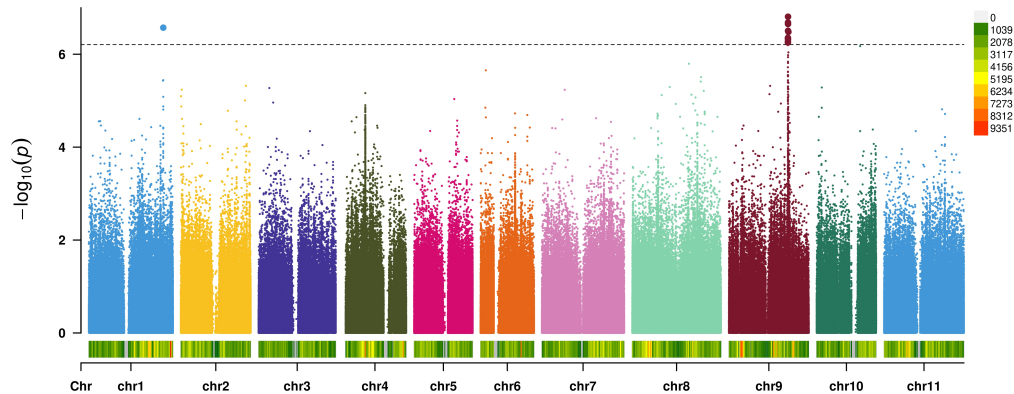

## DF.MLM

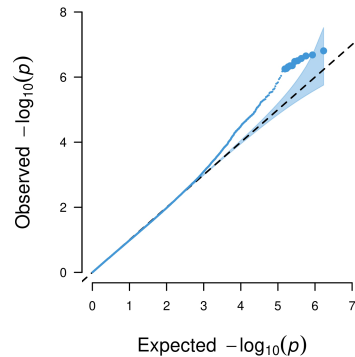

Supplement: Web_Material_uhae201 [file web_material_uhae201.zip › Figure S8.pdf]

## Days to 75% maturity (DM)

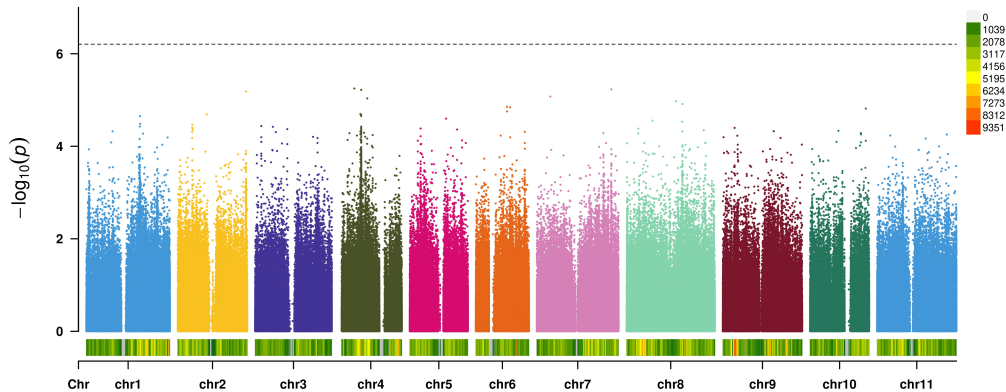

## DM.MLM

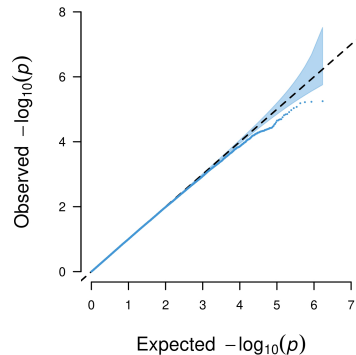

Supplement: Web_Material_uhae201 [file web_material_uhae201.zip › Figure S9.pdf]

## Primary branches per plant (PBPP)

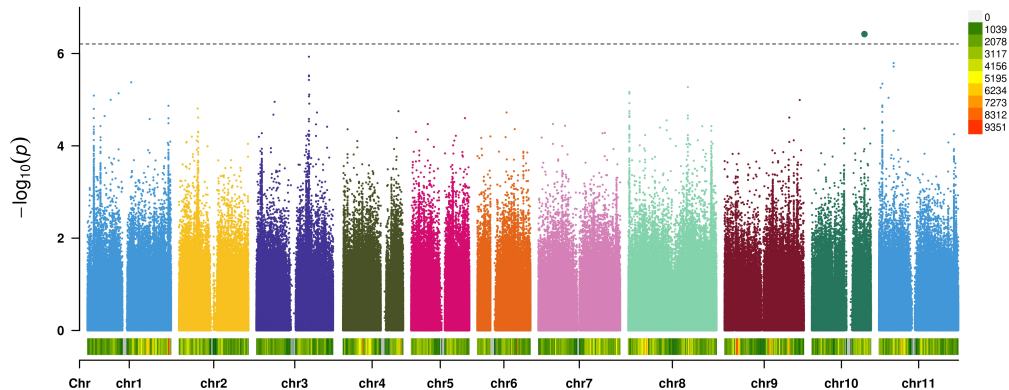

## PBPP.MLM

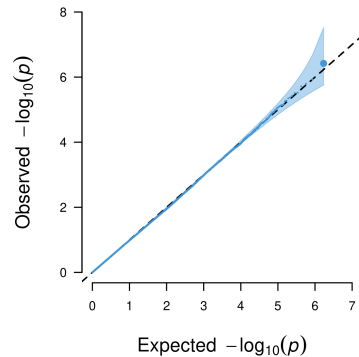

Supplement: Web_Material_uhae201 [file web_material_uhae201.zip › Figure S10.pdf]

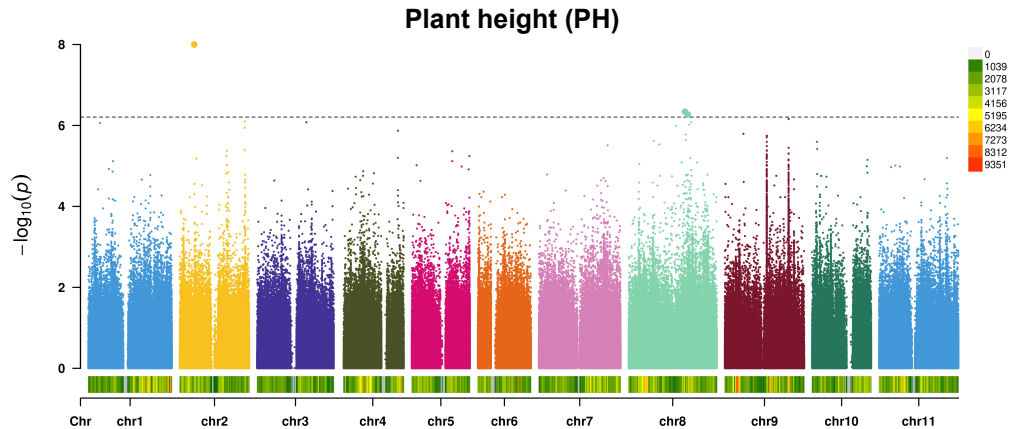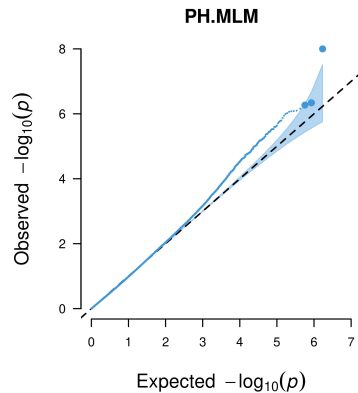

Supplement: Web_Material_uhae201 [file web_material_uhae201.zip › Figure S11.pdf]

Pods per plant (PODPP)

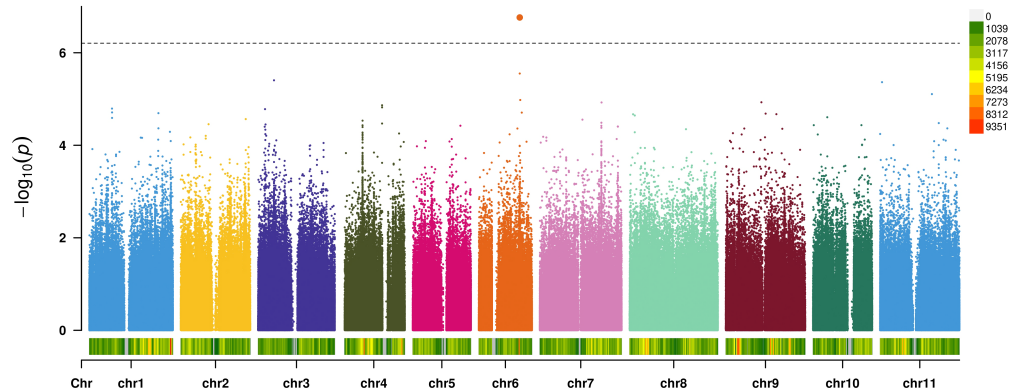

PODPP.MLM

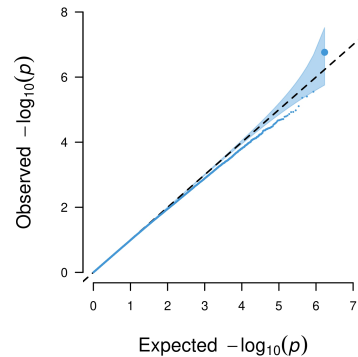

Supplement: Web_Material_uhae201 [file web_material_uhae201.zip › Figure S12.pdf]

## Secondary branches per plant (SBPP)

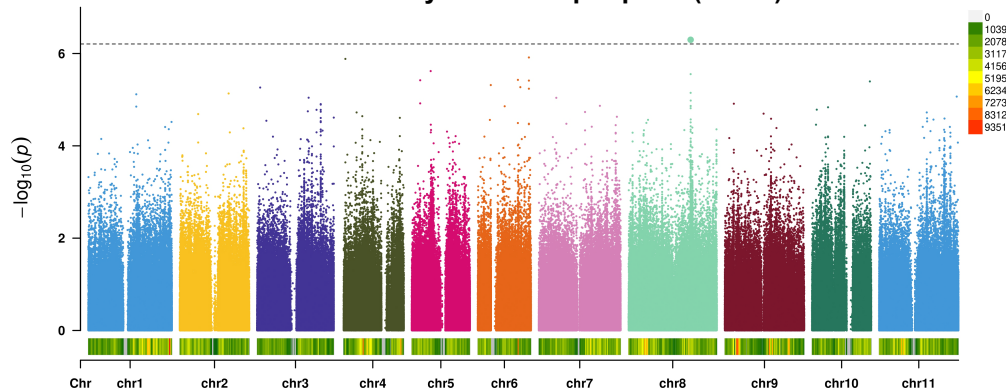

## SBPP.MLM

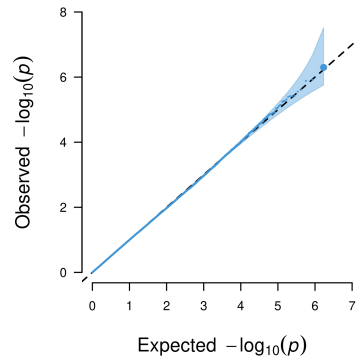

Supplement: Web_Material_uhae201 [file web_material_uhae201.zip › Figure S13.pdf]

## Seeds per plant (SEEDPP)

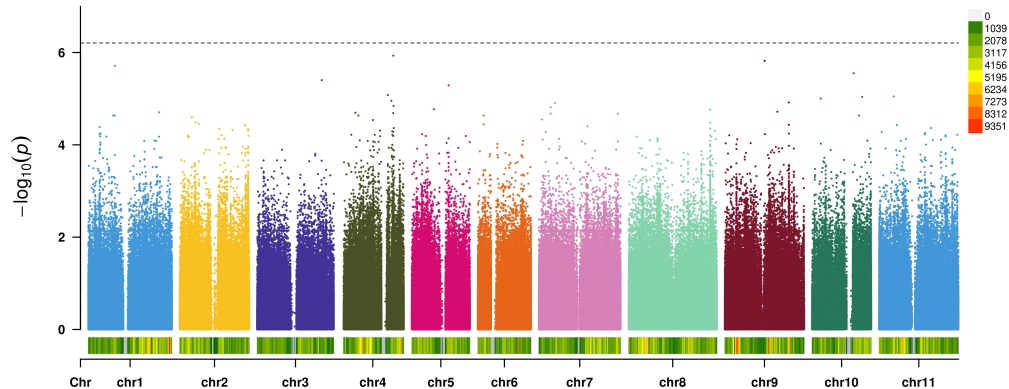

## SEEDPP.MLM

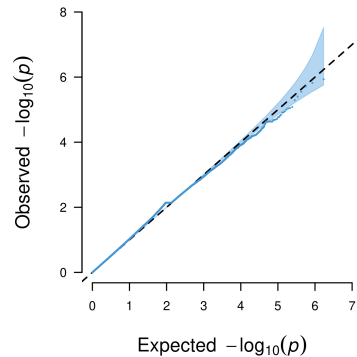

Supplement: Web_Material_uhae201 [file web_material_uhae201.zip › Figure S14.pdf]

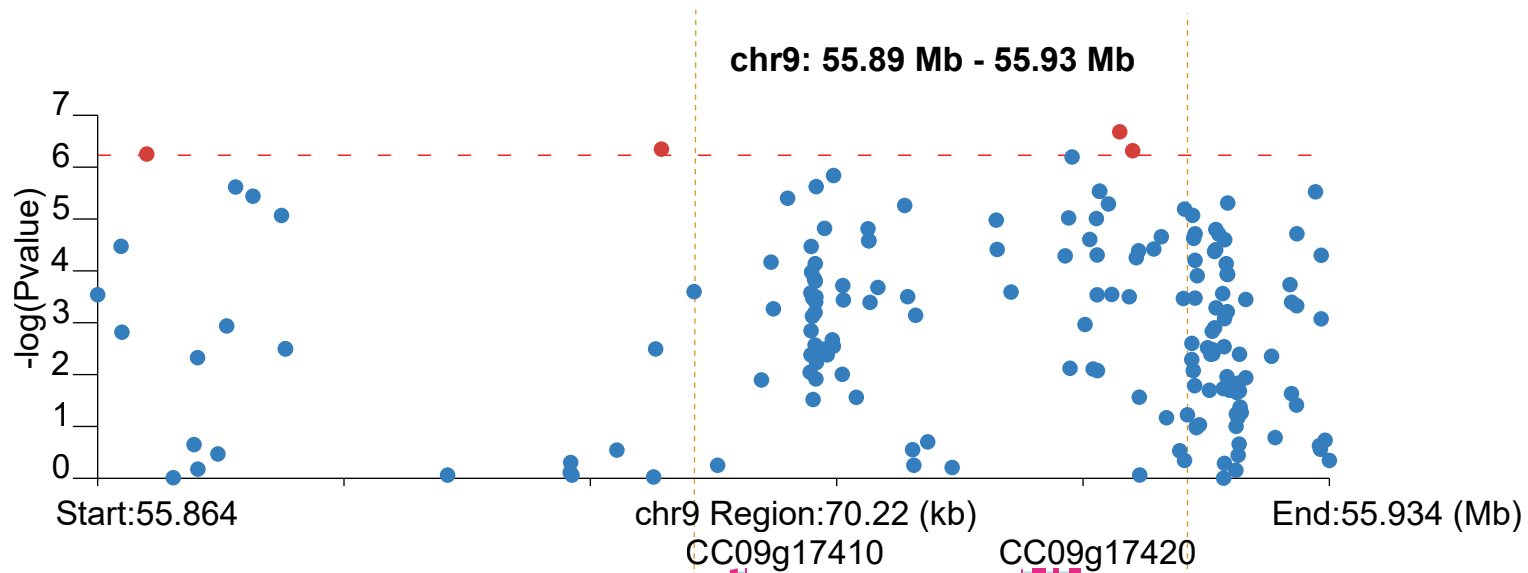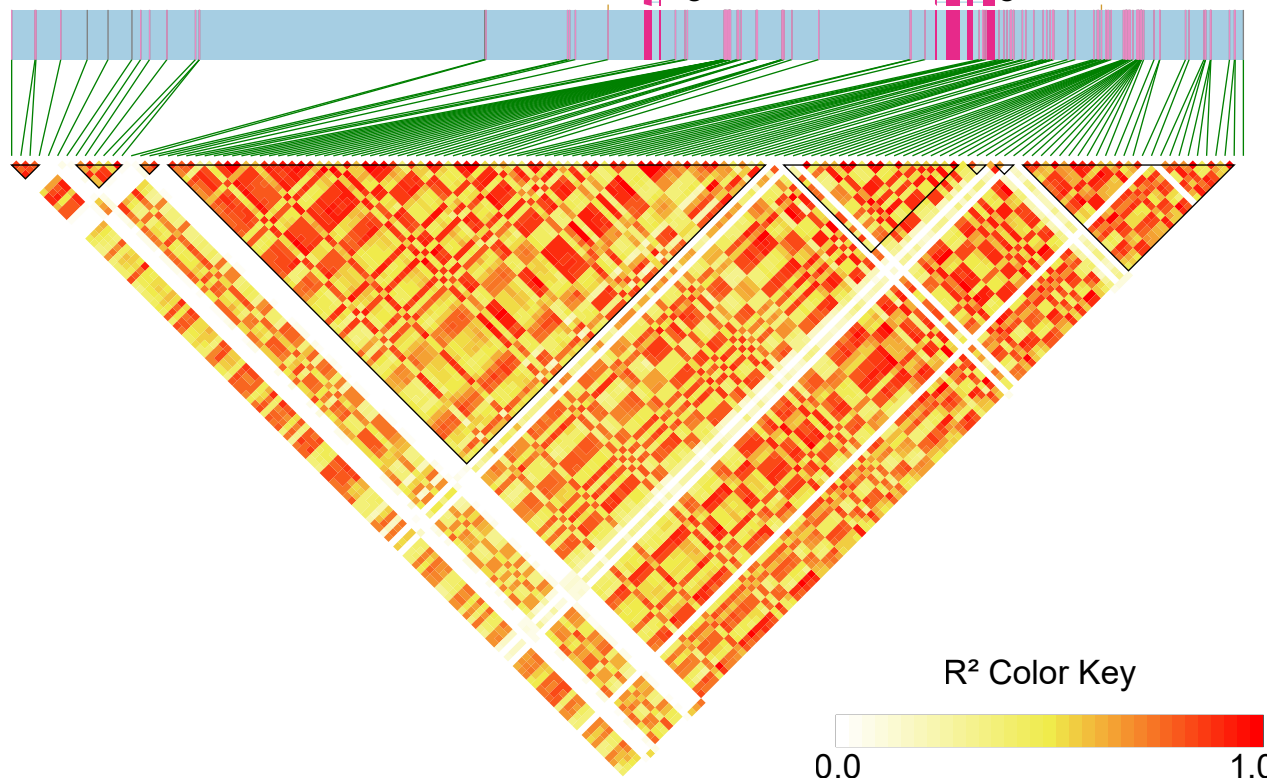

Supplement: Web_Material_uhae201 [file web_material_uhae201.zip › Figure S15.pdf]

Days to 50% flowering (DF)

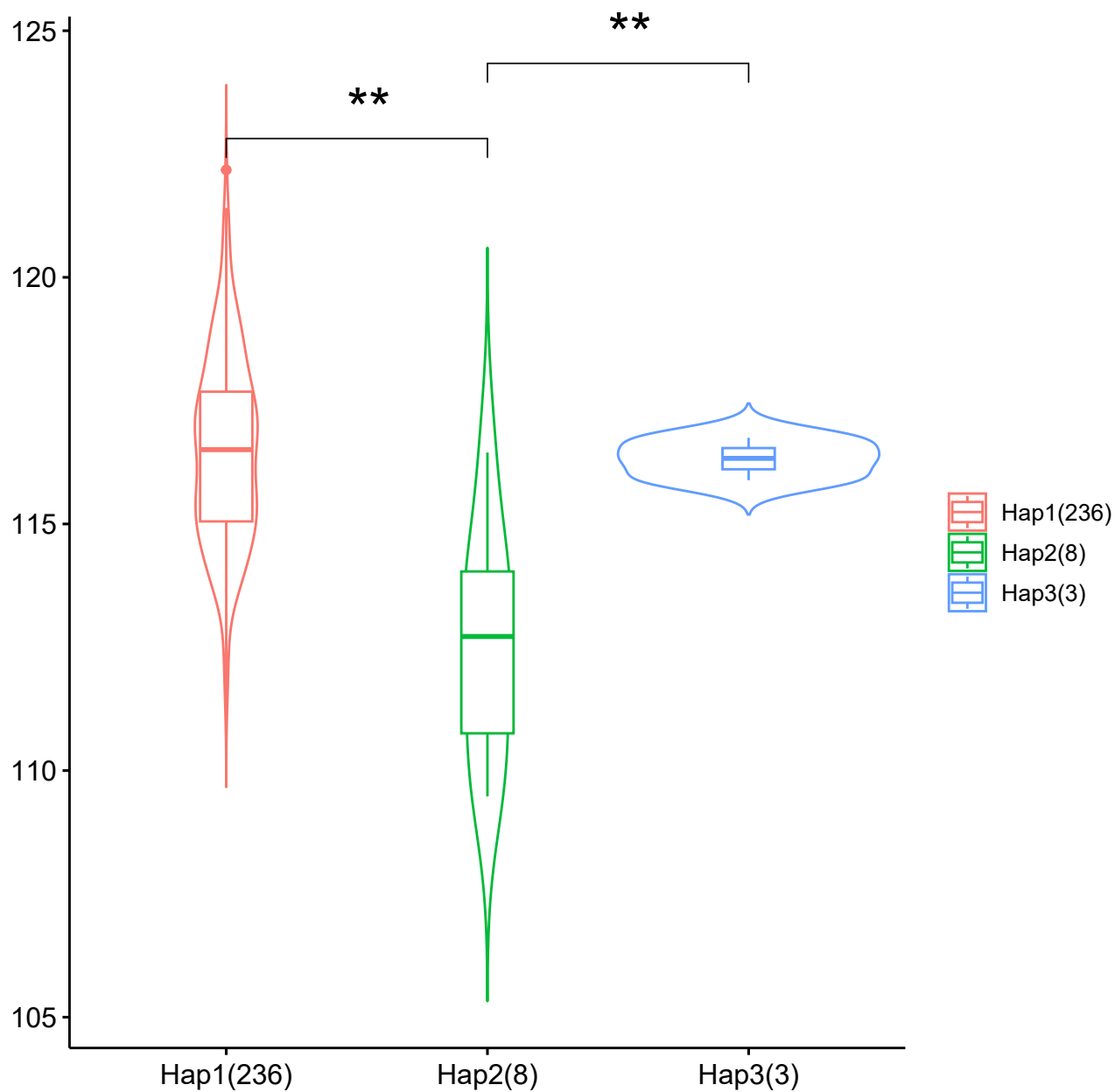

Supplement: Web_Material_uhae201 [file web_material_uhae201.zip › Figure S17.pdf]
